# Arthropoda; Crustacea; Decapoda of deep-sea volcanic habitats of the Galapagos Marine Reserve, Tropical Eastern Pacific

**DOI:** 10.3897/BDJ.8.e54482

**Published:** 2020-09-03

**Authors:** Camila Arnés-Urgellés, Salome Buglass, Shane T. Ahyong, Pelayo Salinas-de-León, Mary K. Wicksten, Leigh Marsh

**Affiliations:** 1 Charles Darwin Research Station, Charles Darwin Foundation, Av. Charles Darwin s/n, Puerto Ayora, Galapagos, Ecuador Charles Darwin Research Station, Charles Darwin Foundation, Av. Charles Darwin s/n, Puerto Ayora Galapagos Ecuador; 2 Australian Museum Research Institute, 1 William St., Sydney, NSW 2010, Australia and School of Biological, Earth and Environmental Sciences, University of New South Wales, Kensington, Sydney, Australia Australian Museum Research Institute, 1 William St., Sydney, NSW 2010, Australia and School of Biological, Earth and Environmental Sciences, University of New South Wales, Kensington Sydney Australia; 3 Pristine Seas, National Geographic Society, Washington, D.C., United States of America Pristine Seas, National Geographic Society Washington, D.C. United States of America; 4 Department of Biology, Texas A&M University, College Station, Texas, United States of America Department of Biology, Texas A&M University, College Station Texas United States of America; 5 Ocean and Earth Science, University of Southampton, Waterfront Campus, Southampton, United Kingdom Ocean and Earth Science, University of Southampton, Waterfront Campus Southampton United Kingdom

**Keywords:** Deep-sea, Galapagos Marine Reserve, macroinvertebrate, Arthropoda, seamounts, volcanic seascapes

## Abstract

**Background:**

The deep-sea biome (> 200 m depth) is the world’s last great wilderness, covering more than 65% of the earth’s surface. Due to rapid technological advances, deep-sea environments are becoming more accessible to scientific research and ocean exploration around the world and, in recent years, this is also true for the Galapagos Islands. Deep-sea habitats cover the largest proportion of Galapagos Marine Reserve (GMR), yet to date, no comprehensive baseline exists on the biodiversity of the benthic fauna associated with volcanic seafloor formations within this region. Closing this knowledge gap is essential to provide information for decision-making for the management of marine resources within the GMR and assessing any potential changes in biodiversity resulting from climate-driven alterations that deep-sea environments are expected to experience. In 2015, the Charles Darwin Foundation’s Seamounts of the GMR Research Project, together with the Galapagos National Park Directorate (GNPD) and Ocean Exploration Trust (OET), conducted a joint expedition on board the EV *Nautilus*. Using Remotely operated vehicles (ROVs), the aim of the expedition was to characterise the geological formations and biological communities present on seamounts, lava flows and other deep-sea habitats (> 200 m) within the GMR.

**New information:**

We provide the first comprehensive image inventory for the phylum Arthropoda from 260 to 3400 m of depth within the GMR. Past studies on deep-sea macroinvertebrates in the GMR have been limited to voucher samples collected from dredging (restricted to soft bottom environments) or by submersibles (only allowing limited biological sampling). The image inventory, presented here, is based on high-definition video transects conducted by the Hercules ROV on board the EV *Nautilus*. Images of macroinvertebrate morphospecies were captured, catalogued and identified, thus providing the first known image inventory of in-situ macroinvertebrate species from the deep-sea region of the GMR.

We present 32 distinct morphospecies occurrences within the class Malacostraca and order Decapoda. We also report 17 different families, three species that are new records to the GMR, in-situ images of two new species to science recently described and one possible new squat lobster, as well as interesting behavioural observations.

## Introduction

The Galapagos archipelago is a volcanic island chain located in the Tropical Eastern Pacific, south of the Galapagos Spreading Center and forms part of the western end of the Carnegie Ridge (White et al. 1993). The archipelago originated approximately 10 Myr from the active volcanism of a melting anomaly (or “hotspot”) in the Earth’s upper mantle in the eastward-moving Nazca Plate (Glass et al. 2007). Resulting from this region’s active volcanic history, both the terrestrial and marine environments of the archipelago are defined by complex and diverse lava formations (Sinton et al. 1996, Hoernle et al. 2000), with the seafloor on and around the Galapagos platform being a heterogeneous seascape shaped by seamounts, lava flows, slopes, rift zones and hydrothermal vents (Geist et al. 2008). Therefore, these environments are likely to host rich deep-sea invertebrate communities (McClain and Barry 2010, Souza Júnior et al. 2014). To date, however, relatively little is known about the life and physical environments of these deep ocean seascapes compared to their shallow water counterparts.

**Figure 1.**
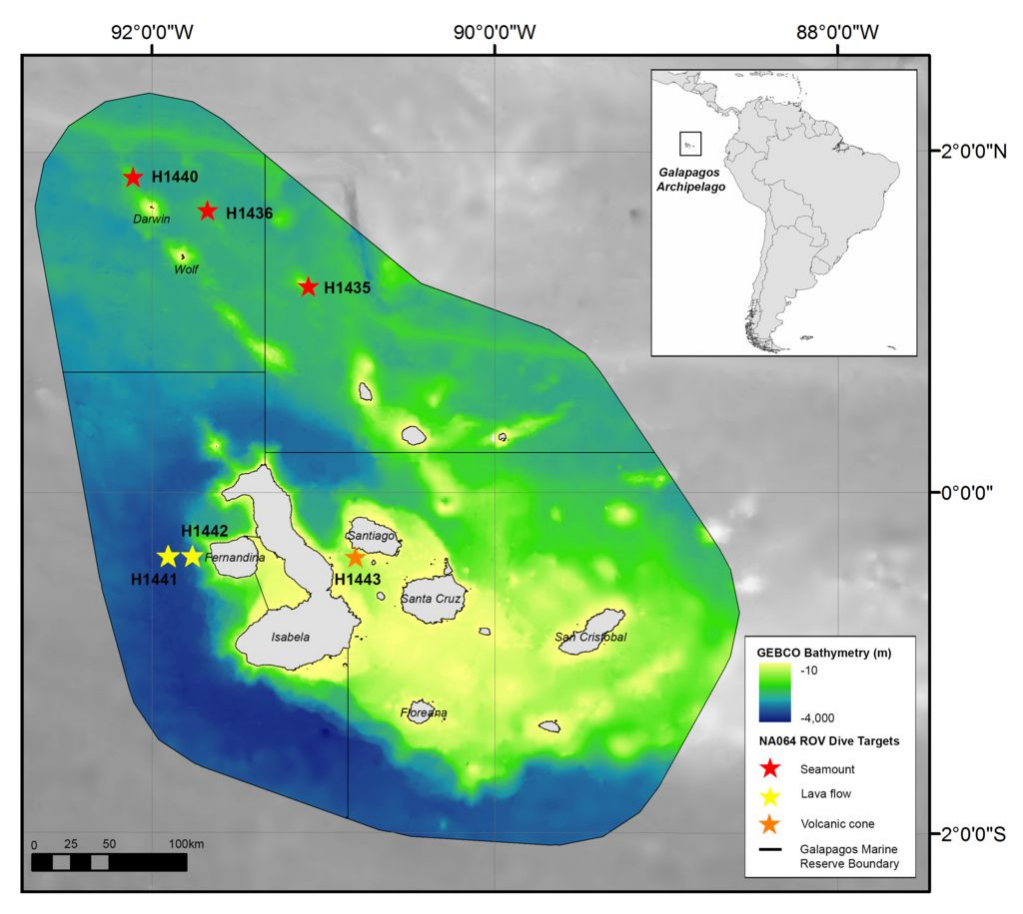
Map of the Galapagos Marine Reserve with the EV *Nautilus* dives locations.

The Galapagos Marine Reserve (GMR) covers approximately 138,000 km^2^ and was established in 1998 (Fig. 1) to protect the archipelago’s marine biodiversity by banning large-scale commercial exploitation of marine resources (Henry and Roberts 2017). While deep-sea environments cover by far the largest proportion of the GMR, the technological challenges of studying deep waters mean that most baseline studies on biodiversity are limited to coastal and shallow pelagic ecosystems (Glynn and Wellington 1983, James 1991). However, there are notable historical exceptions. In the late 1890s, the *Albatross* Expedition pioneered the collection and study of deep-sea fauna in the Tropical Eastern Pacific (Agassiz 1905). Employing dredging methods, benthic sampling was mostly unsuccessful owing to the volcanic nature of the seafloor in the region. Still, some samples were retrieved, but only for soft bottom environments (Bergh and Agassiz 1894, Grove and Lavenberg 1997). Nearly one hundred years later, in 1986, the Harbor Branch Oceanographic Institutes submersible, *Johnson SeaLink*, collected the first targeted collections at a maximum depth of 915 m (Pomponi et al. 1988), with subsequent expeditions in the following years (Banks et al. 2014). Since these expeditions, research efforts have been made to list and describe the biodiversity of Galapagos’ deep-sea environment (Agassiz 1905, Pomponi et al. 1988, Manning 2016), especially on cnidarians (Cairns 1991, Cairns 2018), chordates (McCosker and Smith 1997, Grove and Lavenberg 1997, Barnett et al. 2006, McCosker et al. 2012), echinoderms (Pawson and Ahearn 2000) and isopods (Faxon 1895, Wetzer 1990). Nonetheless, to date, there are no comprehensive image inventories of the deep-sea macrofauna within this region.

In recent decades, the use of Remotely operated vehicles (ROVs), equipped with effective sampling gear and high-resolution recording technologies, has greatly accelerated exploration and surveying of deep-sea habitats (Danovaro et al. 2014) and the use of such deep-submergence technology has contributed to our knowledge of deep-water decapod crustacea in both the western (Komai and Tsuchida 2014) and southern (Poupin et al. 2012) Pacific. In the same way, the *Nautilus* expedition conducted in 2015 marks the future of deep-sea exploration in the GMR and we hope that this inventory, based on in-situ images of the Arthropoda, will facilitate the standardisation of the morphospecies and will be useful for targeted specimen collections during future deep-sea studies in the GMR and the Eastern Tropical Pacific.

Being the first organisms to colonise the islands, even before the appearance of macroscopic plants, the terrestrial arthropods from the Galapagos Islands have been a subject of interest for many years (Peck 1990, Peck 1994). By studying these terrestrial organisms, ecologists and geologists gain a better understanding on how the terrestrial environment of the islands came into being at its present state (Peck et al. 1998). There were intensive collections of arthropods in the intertidal and shallow subtidal regions of the Galapagos Islands during 1931-1938, performed by the ship *Velero III* of the Allan Hancock Foundation, University of Southern California (Meredith and Hancock 1939). These collections resulted in work on brachyurans by Garth (1946), Garth (1991) and records of carideans in various taxonomic publications. James (1991) published a series of accounts of various Galapagos invertebrates but these also were from shallow waters. However, after the initial descriptions in the 1890s of the collections of the *Albatross* Expediton (Faxon 1893, Faxon 1895), deep-sea marine arthropods of the Galapagos received very little attention, compared to their terrestrial and shallow-water counterparts.

## Materials and methods

**Study sites**

In June 2015, the EV *Nautilus* conducted a 10-day collaborative research expedition (NA064) between the Ocean Exploration Trust (OET), the Charles Darwin Foundation (CDF) and the Galapagos National Park Directorate (GNPD) to explore the deep-sea environments of the GMR. All methods were carried out in accordance with relevant guidelines and regulations by the GNPD under research permits PC-26-15 & PC-45-15. All experimental protocols were reviewed and approved by a GNPD’s committee which evaluates animal care during research activities. We conducted a total of six exploratory dives to the far north, west and central part of the Galapagos archipelago (Fig. 1, Table 1).

ROV dives began at the base of each feature and conducted a general upslope transect, following sonar and visual surveys along this transect. Dives H1435, H1436 and H1440 explored three seamounts around the most northern islands of the archipelago, which are part of the Wolf-Darwin volcanic lineament that extends to the Galapagos Spreading Center (Harpp and Geist 2002). All seamounts, located in this area, are conically shaped with small summit craters and relatively flat tops. These are also the youngest seamounts of the Galapagos platform estimated to be less than 1 million years old (Sinton et al. 1996, Harpp and Geist 2002). The deepest ROV transects were conducted during dives H1441 and H1442, which targeted the lava flows and abyssal plains to the west of Fernandina Island. These lava flows are part of the hotspot found beneath the Island where the largest and most active volcano of the Galapagos platform is located (Sinton et al. 1996).

The final dive, H1443, explored two small conically-shaped shallow seamounts located in the central part of the Archipelago, between the islands of Santiago and Isabela. Seamounts from this part of the platform were once centred over the hotspot and are estimated to be between 5 to 6 million years old (Sinton et al. 1996, Harpp and Geist 2002).

**ROV operations**

Seafloor exploration was carried out using the two-body ROV system, Argus and Hercules, each rated to 4000 m water depth. Video and still images of the sites were taken using "Insite Pacific Zeus Plus" HD colour video cameras on both vehicles, each equipped with a 10× mechanical zoom lens. All in-situ images used for the inventory were obtained by Hercules’ mounted camera system. Additionally, environmental parameters were also recorded using Hercules’ telemetry sensors, which included oxygen concentration (Aanderaa Oxygen Optode 3830), temperature and salinity (Seabird FastCat 49Plus).

While the majority of the species analysed for this study were identified from image only, a few specimens were opportunistically collected using the ROV’s hydraulic manipulators. After recovery of the ROV, the collected specimens were preserved following standardised protocols and this information is specified in the 'preparations' and 'notes' sections for each organism listed on the species checklist below.

**Video transects image analysis**

Each ROV dive ranged in duration from 11 to 18 hours. For the subsequent review of morphospecies, each dive was spilt into 2-hour segments. In-situ images of organisms were captured and extracted from video transects analyses using VLC software (Version 3.0.4) by “non-expert analysts”. To avoid reviewer bias in capturing unique morphospecies, five “non-expert analysts” were assigned random video segments from all six ROV dives. Only organisms that appear to be larger than 3 cm were captured and considered for further identification. All images were then tentatively classified under their common names (i.e. squat lobsters, crabs, shrimps, etc.) and only images that appeared in sufficient detail to be determined beyond phyla were sent to taxonomic experts for further identification. Taxonomists identified all images to the lowest taxonomic level possible. To identify the species, taxonomists consulted published literature (e.g. Wicksten 1989) to find out which species were previously reported in the vicinity of the Galapagos Islands. Images were compared to specimens (where available) or photographs with existing illustrations and images from monographs, species descriptions or expedition reports. Many identifications remain tentative because the characteristic features of a species are on the hidden ventral surface or are too small to see in the photograph.

The open nomenclature identification qualifiers presented here are modified from Sigovini et al. (2016). These qualifiers are being developed as part of an initiative by the National Oceanography Centre, Southampton, UK (Dr Tammy Horton, pers. Comm.), to standardise taxonomic nomenclature for image-based faunal analyses.

Below is a brief overview of each qualifier assigned to the different Arthropoda morphospecies. We assigned the qualifier, based on the original comments provided by each taxonomist.

***indet. (indeterminabilis)*** The sign 'indet.' is to be used as an abbreviation of indeterminabilis and to indicate that the specimen is indeterminable beyond a certain taxonomic level due to the lack of diagnostic characters visible in the image. This qualifier can also be used at higher taxonomic ranks and in conjunction with inc. (below) to indicate a difference between the uncertainties of the IDs at higher taxonomic ranks. We also used this term for some of the very poor-quality images.

***inc. (incerta)*** the usage of this qualifier is to be restricted to the meaning of 'uncertain identification' and to be equated to the question mark. Since the latter may be considered as a 'wildcard' by some software, in data stored in digital form, it may be substituted by 'sp. inc.', 'gen. inc.' etc.

***stet. (stetit)*** Use the term stetit after the taxon name to explicitly express the identifier choice of not proceeding further.

A total of 32 distinct morphospecies of arthropods were identified, belonging to 17 families, 19 genera and 13 confirmed species of the class Malacostraca, order Decapoda.

The occurrence dataset presented here can also be found at Deep-sea OBIS node https://doi.org/10.15468/szdxtb via GBIF.org

## Checklists

### Systematic account of Arthropoda species from the Galapagos Marine Reserve; EV *Nautilus* NA064 Expedition 2015

#### Dendrobranchiata

Bate, 1888

E498D42B-DE65-5693-A205-FAFC9C3FFC64

#### Penaeoidea

Rafinesque, 1815

4589412F-320B-57F5-B911-E192ACB79AB0

##### Materials

- **Type status:** Other material. **Occurrence:** recordedBy: CDF Volunteer; behavior: in water column; occurrenceStatus: present; preparations: Image only; associatedMedia: https://farm2.staticflickr.com/1964/44925955814_f60b782df6_o.png; occurrenceID: H1441_160738_Penaeoidea_fam_indet.; **Taxon:** scientificNameID: urn:lsid:marinespecies.org:taxname:106683; scientificName: Penaeoidea; kingdom: Animalia; phylum: Arthropoda; class: Malacostraca; order: Decapoda; scientificNameAuthorship: Rafinesque, 1815; taxonomicStatus: accepted; **Location:** locationID: MRGID8403; waterBody: Pacific Ocean; country: Ecuador; stateProvince: Galapagos; locality: West; verbatimLocality: West of Fernandina; minimumDepthInMeters: 3397; maximumDepthInMeters: 3397; decimalLatitude: -0.3921; decimalLongitude: -91.8793; geodeticDatum: WGS84; coordinateUncertaintyInMeters: 100; **Identification:** identifiedBy: Mary Wicksten; dateIdentified: 2017; identificationRemarks: ID from imagery only; identificationQualifier: Penaeoidea fam. indet.; **Event:** eventID: NA064; samplingProtocol: Remotely Operated Vehicles; eventDate: 07-03-15; eventTime: 4:07:38 PM; habitat: Lava Flow; **Record Level:** language: en; bibliographicCitation: WoRMS (2020). Penaeoidea Rafinesque, 1815. Accessed at: http://www.marinespecies.org/aphia.php?p=taxdetails&id=106683 on 2020-07-08; institutionCode: CDF; collectionCode: Arthropoda; datasetName: Video transect framegrabs; basisOfRecord: HumanObservation

##### Notes

**Figure 2.**
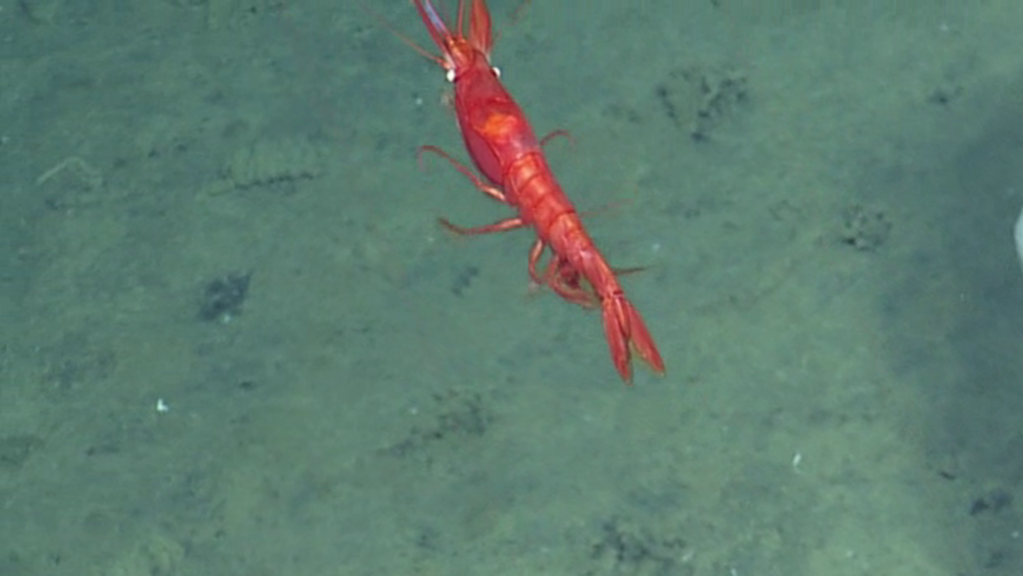
Penaeoidea fam. indet.

This shrimp could belong to the genus *Benthesicymus* (Benthesicymidae) or perhaps a shrimp of the family Aristeidae. It has a short rostrum with teeth and elongate pleopods. The image shows possible white corneas, seen previously amongst the Benthesicymidae. It is not possible to tell if the shrimp in the photograph has true white eyes or the white colour is a reflection from the strobe light. Fig. 2

#### Pleocyemata

Burkenroad, 1963

33E47C69-B86A-5951-AC18-7183CB60F5E3

#### Anomura

Macleay, 1838

8117491D-0B0A-5CD6-8B00-A1FCE52D004A

#### Chirostyloidea

Ortmann, 1892

7B665EF4-14E4-520F-A57C-2FC900E6ECA1

#### Heteroptychus nautilus

Baba & Wicksten, 2019

115735E6-8276-5A69-82E1-42DD11FDA3D7

##### Materials

- **Type status:** Other material. **Occurrence:** recordedBy: CDF Volunteer; behavior: associated with octocorallia; occurrenceStatus: present; preparations: Image | 75% ETOH; associatedMedia: https://farm2.staticflickr.com/1980/45600650492_d3cc781b0d_o.png; occurrenceID: H1435_023826_Heteroptychus_nautilus; **Taxon:** scientificNameID: urn:lsid:marinespecies.org:taxname:1332462; scientificName: Heteroptychus nautilus; kingdom: Animalia; phylum: Arthropoda; class: Malacostraca; order: Decapoda; family: Chirostylidae; genus: Heteroptychus; specificEpithet: nautilus; scientificNameAuthorship: Baba & Wicksten, 2019; taxonomicStatus: accepted; **Location:** locationID: MRGID8403; waterBody: Pacific Ocean; country: Ecuador; stateProvince: Galapagos; locality: North; verbatimLocality: East of Wolf; minimumDepthInMeters: 1049; maximumDepthInMeters: 1049; decimalLatitude: 1.2157; decimalLongitude: -91.0924; geodeticDatum: WGS84; coordinateUncertaintyInMeters: 30; **Identification:** identifiedBy: Shane Ahyong | Keiji Baba; dateIdentified: 2017; identificationRemarks: Image ID confirmed from morphology; identificationQualifier: Heteroptychus nautilus; **Event:** eventID: NA064; samplingProtocol: Remotely Operated Vehicles; eventDate: 06-27-15; eventTime: 2:38:26 AM; habitat: Seamount; **Record Level:** language: en; bibliographicCitation: WoRMS (2019). Heteroptychus nautilus Baba & Wicksten, 2019. Accessed at: http://www.marinespecies.org/aphia.php?p=taxdetails&id=1332462 on 2019-08-23; institutionCode: CDF; collectionCode: Arthropoda; datasetName: Video transect framegrabs; basisOfRecord: HumanObservation

##### Notes

**Figure 3.**
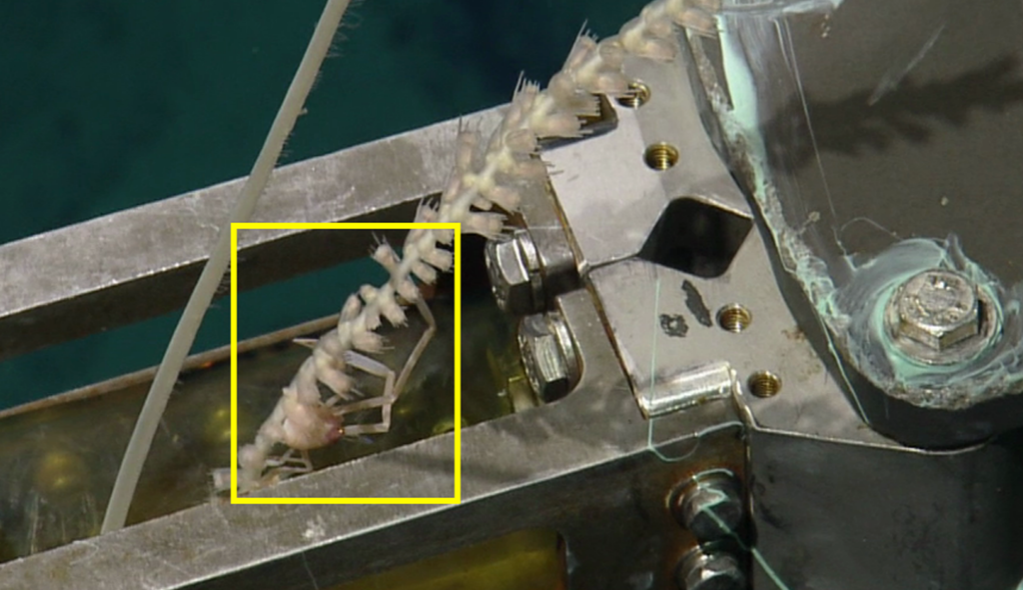
*Heteroptychus nautilus*

This is an image of the holotype being sampled. Described in Baba and Wicksten (2019) (Sample NA064-009-01-01-A). Fig. 3

#### Uroptychus bellus

Faxon, 1893

9BE46CA8-3F7C-5E19-9B37-A3F473B287E5

##### Materials

- **Type status:** Other material. **Occurrence:** recordedBy: CDF Volunteer; behavior: associated with antipatharia; occurrenceStatus: present; preparations: Image | 75% ETOH; associatedMedia: https://farm2.staticflickr.com/1901/43833022820_8a5e3d5686_o.png; occurrenceID: H1443_034319_Uroptychus_sp_inc_bellus; **Taxon:** scientificNameID: urn:lsid:marinespecies.org:taxname:392056; scientificName: Uroptychus bellus; kingdom: Animalia; phylum: Arthropoda; class: Malacostraca; order: Decapoda; family: Chirostylidae; genus: Uroptychus; specificEpithet: bellus; scientificNameAuthorship: Faxon, 1893; taxonomicStatus: accepted; **Location:** locationID: MRGID8403; waterBody: Pacific Ocean; country: Ecuador; stateProvince: Galapagos; locality: Southeast; verbatimLocality: Galapagos Platform; minimumDepthInMeters: 473; maximumDepthInMeters: 473; decimalLatitude: -0.3804; decimalLongitude: -90.8179; geodeticDatum: WGS84; coordinateUncertaintyInMeters: 15; **Identification:** identifiedBy: Shane Ahyong | Mary Wicksten; dateIdentified: 2017; identificationRemarks: Image ID confirmed from morphology; identificationQualifier: Uroptychus bellus; **Event:** eventID: NA064; samplingProtocol: Remotely Operated Vehicles; eventDate: 07-06-15; eventTime: 3:43:19 AM; habitat: Volcanic Cone; **Record Level:** language: en; bibliographicCitation: WoRMS (2019). Uroptychus bellus Faxon, 1893. Accessed at: http://www.marinespecies.org/aphia.php?p=taxdetails&id=392056 on 2019-08-23; institutionCode: CDF; collectionCode: Arthropoda; datasetName: Video transect framegrabs; basisOfRecord: HumanObservation

##### Notes

**Figure 4.**
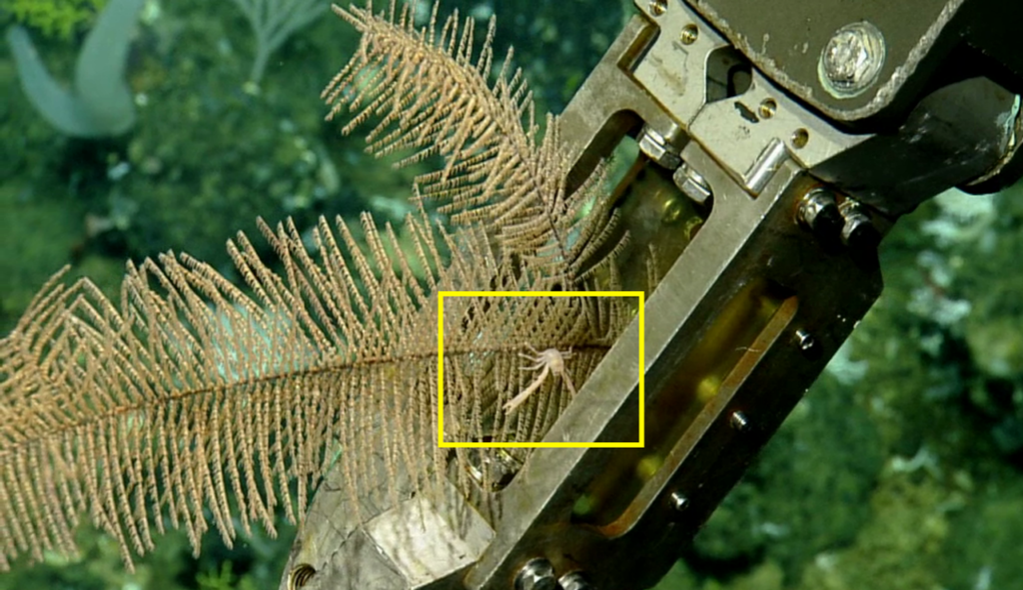
*Uroptychus bellus*

*Uroptychus bellus* from morphology (Sample NA064-130-01-01-A). Described in Baba and Wicksten (2019). Fig. 4

#### Uroptychus

Henderson, 1888

77D095C7-12A8-5AF4-B857-ACBBDE6305FB

##### Materials

- **Type status:** Other material. **Occurrence:** recordedBy: CDF Volunteer; behavior: associated with octocorallia; occurrenceStatus: present; preparations: Image only; associatedMedia: https://farm2.staticflickr.com/1925/45600648622_c59bfa2e89_o.png; occurrenceID: H1435_071407_Uroptychus_sp_inc_compressus; **Taxon:** scientificNameID: urn:lsid:marinespecies.org:taxname:106833; scientificName: Uroptychus; kingdom: Animalia; phylum: Arthropoda; class: Malacostraca; order: Decapoda; family: Chirostylidae; genus: Uroptychus; scientificNameAuthorship: Henderson, 1888; taxonomicStatus: accepted; **Location:** locationID: MRGID8403; waterBody: Pacific Ocean; country: Ecuador; stateProvince: Galapagos; locality: North; verbatimLocality: East of Wolf; minimumDepthInMeters: 812; maximumDepthInMeters: 812; decimalLatitude: 1.2243; decimalLongitude: -91.1028; geodeticDatum: WGS84; coordinateUncertaintyInMeters: 25; **Identification:** identifiedBy: Shane Ahyong; dateIdentified: 2017; identificationRemarks: ID from imagery only; identificationQualifier: *Uroptychus compressus* sp. inc.; **Event:** eventID: NA064; samplingProtocol: Remotely Operated Vehicles; eventDate: 06-27-15; eventTime: 7:14:07 AM; habitat: Seamount; **Record Level:** language: en; bibliographicCitation: WoRMS (2019). Uroptychus Henderson, 1888. Accessed at: http://www.marinespecies.org/aphia.php?p=taxdetails&id=106833 on 2019-08-23; institutionCode: CDF; collectionCode: Arthropoda; datasetName: Video transect framegrabs; basisOfRecord: HumanObservation

##### Notes

**Figure 5.**
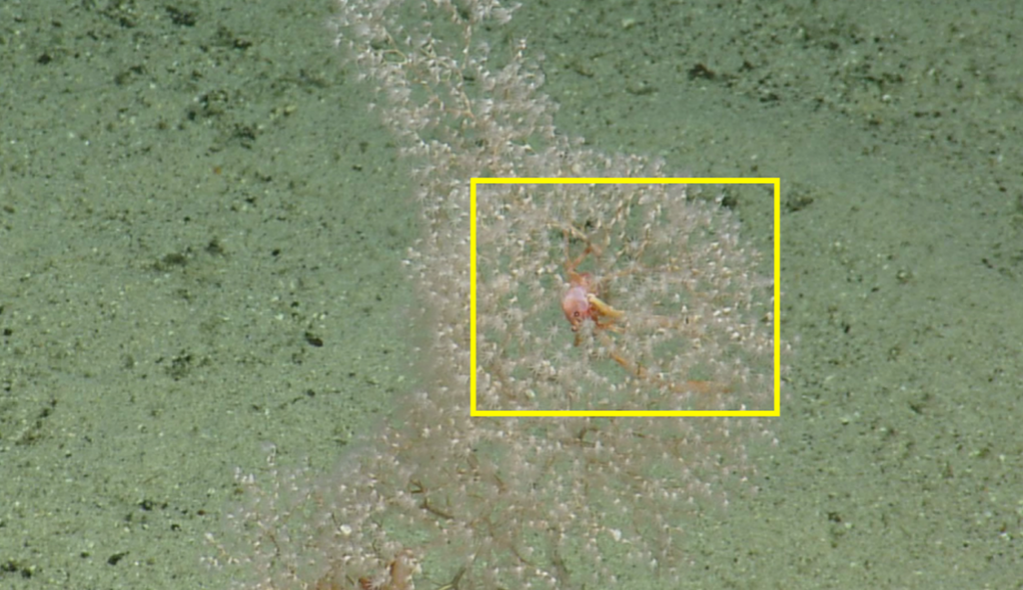
*Uroptychus compressus* sp. inc.

Species of *Uroptychus* are characteristic inhabitants of the soft coral *Chrysogorgia. Uroptychus compressus* has been reported in the area of study. Fig. 5

#### Eumunida subsolanus

Baba & Wicksten, 2019

7F0D80C1-559C-510C-9255-C115D9EACA94

##### Materials

- **Type status:** Other material. **Occurrence:** recordedBy: CDF Volunteer; behavior: on sea floor; occurrenceStatus: present; preparations: Image only; associatedMedia: https://farm2.staticflickr.com/1944/43833019450_3f3d6792d3_o.png; occurrenceID: H1435_085733_Eumunida_subsolanus; **Taxon:** scientificNameID: urn:lsid:marinespecies.org:taxname:1332450; scientificName: Eumunida subsolanus; kingdom: Animalia; phylum: Arthropoda; class: Malacostraca; order: Decapoda; family: Eumunididae; genus: Eumunida; specificEpithet: subsolanus; scientificNameAuthorship: Baba & Wicksten, 2019; taxonomicStatus: accepted; **Location:** locationID: MRGID8403; waterBody: Pacific Ocean; country: Ecuador; stateProvince: Galapagos; locality: North; verbatimLocality: East of Wolf; minimumDepthInMeters: 515; maximumDepthInMeters: 515; decimalLatitude: 1.2271; decimalLongitude: -91.1099; geodeticDatum: WGS84; coordinateUncertaintyInMeters: 15; **Identification:** identifiedBy: Shane Ahyong; dateIdentified: 2017; identificationRemarks: ID from imagery only; identificationQualifier: Eumunida subsolanus; **Event:** eventID: NA064; samplingProtocol: Remotely Operated Vehicles; eventDate: 06-27-15; eventTime: 08:57:33 AM; habitat: Seamount; **Record Level:** language: en; bibliographicCitation: WoRMS (2019). Eumunida subsolanus Baba & Wicksten, 2019. Accessed at: http://www.marinespecies.org/aphia.php?p=taxdetails&id=1332450 on 2019-08-23; institutionCode: CDF; collectionCode: Arthropoda; datasetName: Video transect framegrabs; basisOfRecord: HumanObservation
- **Type status:** Other material. **Occurrence:** recordedBy: CDF Volunteer; behavior: associated with hexacorallia; occurrenceStatus: present; preparations: Image only; associatedMedia: https://farm2.staticflickr.com/1936/30710228737_be8e868f29_o.png; occurrenceID: H1443_015727_Eumunida_subsolanus; **Taxon:** scientificNameID: urn:lsid:marinespecies.org:taxname:1332450; scientificName: Eumunida subsolanus; kingdom: Animalia; phylum: Arthropoda; class: Malacostraca; order: Decapoda; family: Eumunididae; genus: Eumunida; specificEpithet: subsolanus; scientificNameAuthorship: Baba & Wicksten, 2019; taxonomicStatus: accepted; **Location:** locationID: MRGID8403; waterBody: Pacific Ocean; country: Ecuador; stateProvince: Galapagos; locality: Southeast; verbatimLocality: Galapagos Platform; minimumDepthInMeters: 444; maximumDepthInMeters: 444; decimalLatitude: -0.3766; decimalLongitude: -90.8176; geodeticDatum: WGS84; coordinateUncertaintyInMeters: 15; **Identification:** identifiedBy: Shane Ahyong; dateIdentified: 2017; identificationRemarks: ID from imagery only; identificationQualifier: Eumunida subsolanus; **Event:** eventID: NA064; samplingProtocol: Remotely Operated Vehicles; eventDate: 07-06-15; eventTime: 1:57:27 AM; habitat: Volcanic Cone; **Record Level:** language: en; bibliographicCitation: WoRMS (2019). Eumunida subsolanus Baba & Wicksten, 2019. Accessed at: http://www.marinespecies.org/aphia.php?p=taxdetails&id=1332450 on 2019-08-23; institutionCode: CDF; collectionCode: Arthropoda; datasetName: Video transect framegrabs; basisOfRecord: HumanObservation
- **Type status:** Other material. **Occurrence:** recordedBy: CDF Volunteer; behavior: on sea floor; occurrenceStatus: present; preparations: Image only; associatedMedia: https://farm2.staticflickr.com/1948/44926105094_597fb494b2_o.png; occurrenceID: H1443_201511_Eumunida_subsolanus; **Taxon:** scientificNameID: urn:lsid:marinespecies.org:taxname:1332450; scientificName: Eumunida subsolanus; kingdom: Animalia; phylum: Arthropoda; class: Malacostraca; order: Decapoda; family: Eumunididae; genus: Eumunida; specificEpithet: subsolanus; scientificNameAuthorship: Baba & Wicksten, 2019; taxonomicStatus: accepted; **Location:** locationID: MRGID8403; waterBody: Pacific Ocean; country: Ecuador; stateProvince: Galapagos; locality: Southeast; verbatimLocality: Galapagos Platform; minimumDepthInMeters: 421; maximumDepthInMeters: 421; decimalLatitude: -0.3740; decimalLongitude: -90.8150; geodeticDatum: WGS84; coordinateUncertaintyInMeters: 15; **Identification:** identifiedBy: Shane Ahyong; dateIdentified: 2017; identificationRemarks: ID from imagery only; identificationQualifier: Eumunida subsolanus; **Event:** eventID: NA064; samplingProtocol: Remotely Operated Vehicles; eventDate: 07-05-15; eventTime: 20:15:11 PM; habitat: Volcanic Cone; **Record Level:** language: en; bibliographicCitation: WoRMS (2019). Eumunida subsolanus Baba & Wicksten, 2019. Accessed at: http://www.marinespecies.org/aphia.php?p=taxdetails&id=1332450 on 2019-08-23; institutionCode: CDF; collectionCode: Arthropoda; datasetName: Video transect framegrabs; basisOfRecord: HumanObservation
- **Type status:** Other material. **Occurrence:** recordedBy: CDF Volunteer; behavior: on sea floor; occurrenceStatus: present; preparations: Image only; associatedMedia: https://farm2.staticflickr.com/1966/43833012570_3e6c428cf9_o.png; occurrenceID: H1443_201343_Eumunida_subsolanus; **Taxon:** scientificNameID: urn:lsid:marinespecies.org:taxname:1332450; scientificName: Eumunida subsolanus; kingdom: Animalia; phylum: Arthropoda; class: Malacostraca; order: Decapoda; family: Eumunididae; genus: Eumunida; specificEpithet: subsolanus; scientificNameAuthorship: Baba & Wicksten, 2019; taxonomicStatus: accepted; **Location:** locationID: MRGID8403; waterBody: Pacific Ocean; country: Ecuador; stateProvince: Galapagos; locality: Southeast; verbatimLocality: Galapagos Platform; minimumDepthInMeters: 422; maximumDepthInMeters: 422; decimalLatitude: -0.3740; decimalLongitude: -90.8150; geodeticDatum: WGS84; coordinateUncertaintyInMeters: 15; **Identification:** identifiedBy: Shane Ahyong; dateIdentified: 2017; identificationRemarks: ID from imagery only; identificationQualifier: Eumunida subsolanus; **Event:** eventID: NA064; samplingProtocol: Remotely Operated Vehicles; eventDate: 07-05-15; eventTime: 20:13:43 PM; habitat: Volcanic Cone; **Record Level:** language: en; bibliographicCitation: WoRMS (2019). Eumunida subsolanus Baba & Wicksten, 2019. Accessed at: http://www.marinespecies.org/aphia.php?p=taxdetails&id=1332450 on 2019-08-23; institutionCode: CDF; collectionCode: Arthropoda; datasetName: Video transect framegrabs; basisOfRecord: HumanObservation

##### Notes

In-situ images of *Eumunida subsolanus* described in Baba and Wicksten (2019), which is a new species discovered as a result of the NA064 expedition. Fig. 6

#### Sternostylus defensus

(Benedict, 1902)

D4F5028F-757E-50E3-8EBC-1E8086D2B98B

##### Materials

- **Type status:** Other material. **Occurrence:** recordedBy: CDF Volunteer; behavior: associated with antipatharia; occurrenceStatus: present; preparations: Image only; associatedMedia: https://live.staticflickr.com/65535/48419070577_79a7040fb0_o.png; occurrenceID: H1443_022005_Sternostylus_defensus; **Taxon:** scientificNameID: urn:lsid:marinespecies.org:taxname:1310473; scientificName: Sternostylus defensus; kingdom: Animalia; phylum: Arthropoda; class: Malacostraca; order: Decapoda; family: Sternostylidae; genus: Sternostylus; specificEpithet: defensus; scientificNameAuthorship: (Benedict, 1902); taxonomicStatus: accepted; **Location:** locationID: MRGID8403; waterBody: Pacific Ocean; country: Ecuador; stateProvince: Galapagos; locality: Far North; verbatimLocality: Northwest of Darwin; minimumDepthInMeters: 1210; maximumDepthInMeters: 1210; decimalLatitude: 1.8779; decimalLongitude: -92.128; geodeticDatum: WGS84; coordinateUncertaintyInMeters: 35; **Identification:** identifiedBy: Shane Ahyong; dateIdentified: 2017; identificationRemarks: ID from imagery only; identificationQualifier: Sternostylus defensus; **Event:** eventID: NA064; samplingProtocol: Remotely Operated Vehicles; eventDate: 07-02-15; eventTime: 5:35:34 AM; habitat: Seamount; **Record Level:** language: en; bibliographicCitation: WoRMS (2019). Sternostylus defensus (Benedict, 1902). Accessed at: http://www.marinespecies.org/aphia.php?p=taxdetails&id=1310473 on 2019-08-23; institutionCode: CDF; collectionCode: Arthropoda; datasetName: Video transect framegrabs; basisOfRecord: HumanObservation

##### Notes

**Figure 7.**
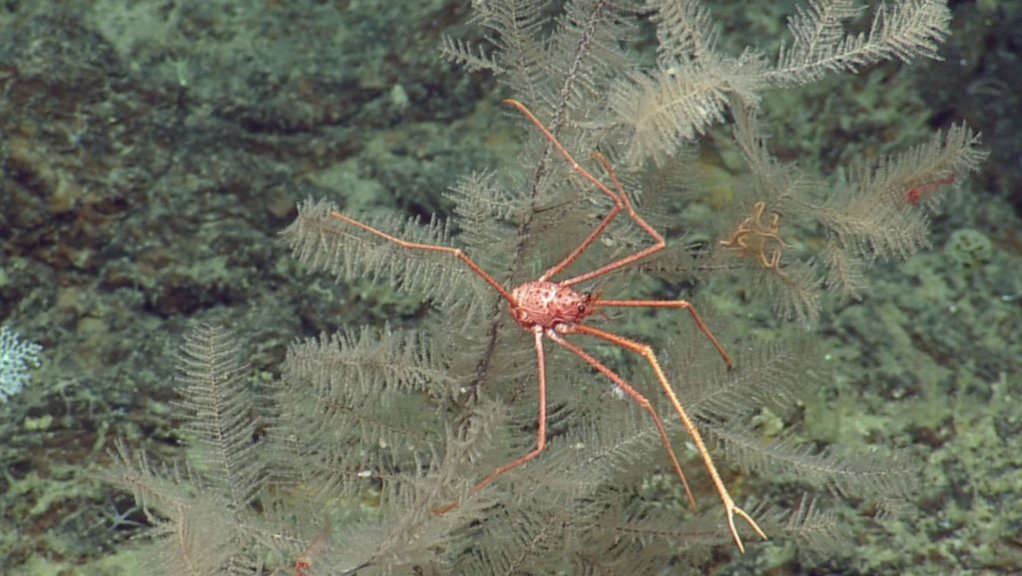
*Sternostylus defensus*

This observation is a new record for Galapagos. Fig. 7

#### Sternostylus

Baba, Ahyong & Schnabel, 2018

53435DED-3E69-5C96-A132-C156C1709F33

##### Materials

- **Type status:** Other material. **Occurrence:** recordedBy: CDF Volunteer; behavior: associated with octocorallia; occurrenceStatus: present; preparations: Image only; associatedMedia: https://farm2.staticflickr.com/1958/45600653072_11699d38c5_o.png; occurrenceID: H1443_014622_Sternostylus_sp_indet.; **Taxon:** scientificNameID: urn:lsid:marinespecies.org:taxname:1310469; scientificName: Sternostylus; kingdom: Animalia; phylum: Arthropoda; class: Malacostraca; order: Decapoda; family: Sternostylidae; genus: Sternostylus; scientificNameAuthorship: Baba, Ahyong & Schnabel, 2018; taxonomicStatus: accepted; **Location:** locationID: MRGID8403; waterBody: Pacific Ocean; country: Ecuador; stateProvince: Galapagos; locality: Southeast; verbatimLocality: Galapagos Platform; minimumDepthInMeters: 445; maximumDepthInMeters: 445; decimalLatitude: -0.3766; decimalLongitude: -90.8176; geodeticDatum: WGS84; coordinateUncertaintyInMeters: 15; **Identification:** identifiedBy: Shane Ahyong; dateIdentified: 2017; identificationRemarks: ID from imagery only; identificationQualifier: *Sternostylus* sp. indet.; **Event:** eventID: NA064; samplingProtocol: Remotely Operated Vehicles; eventDate: 07-06-15; eventTime: 1:46:22 AM; habitat: Volcanic Cone; **Record Level:** language: en; bibliographicCitation: WoRMS (2019). Sternostylus Baba, Ahyong & Schnabel, 2018. Accessed at: http://www.marinespecies.org/aphia.php?p=taxdetails&id=1310469 on 2019-08-23; institutionCode: CDF; collectionCode: Arthropoda; datasetName: Video transect framegrabs; basisOfRecord: HumanObservation

##### Notes

**Figure 8.**
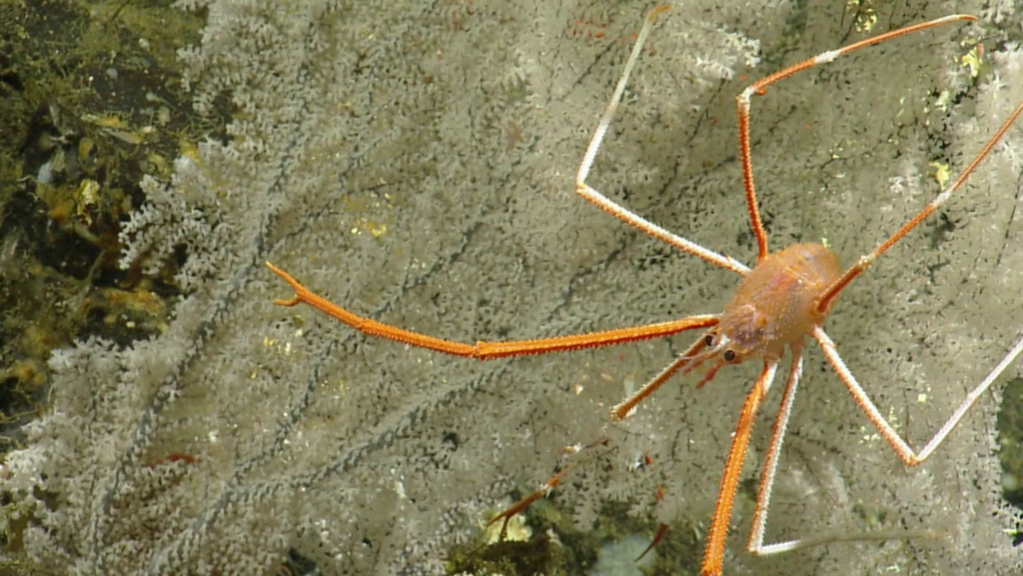
*Sternostylus* sp. indet.

This species is possibly new to science. Fig. 8

#### Galatheoidea

Samouelle, 1819

BE4034EF-8A05-5859-BD9E-F5B7E67263D5

#### Janetogalathea

Baba & Wicksten, 1997

D939CAFB-7A4E-52B8-B89E-CB248EF8B914

##### Materials

- **Type status:** Other material. **Occurrence:** recordedBy: CDF Volunteer; behavior: on seafloor; occurrenceStatus: present; preparations: Image only; associatedMedia: https://farm2.staticflickr.com/1917/45600635422_79c52754ac_o.png; occurrenceID: H1443_225538_Janetogalathea_sp_inc_californiensis; **Taxon:** scientificNameID: urn:lsid:marinespecies.org:taxname:387304; scientificName: Janetogalathea; kingdom: Animalia; phylum: Arthropoda; class: Malacostraca; order: Decapoda; family: Galatheidae; genus: Janetogalathea; scientificNameAuthorship: Baba & Wicksten, 1997; taxonomicStatus: accepted; **Location:** locationID: MRGID8403; waterBody: Pacific Ocean; country: Ecuador; stateProvince: Galapagos; locality: Southeast; verbatimLocality: Galapagos Platform; minimumDepthInMeters: 419; maximumDepthInMeters: 419; decimalLatitude: -0.3723; decimalLongitude: -90.816; geodeticDatum: WGS84; coordinateUncertaintyInMeters: 15; **Identification:** identifiedBy: Shane Ahyong; dateIdentified: 2017; identificationRemarks: ID from imagery only; identificationQualifier: *Janetogalathea californiensis* sp. inc.; **Event:** eventID: NA064; samplingProtocol: Remotely Operated Vehicles; eventDate: 07-05-15; eventTime: 10:55:38 PM; habitat: Volcanic Cone; **Record Level:** language: en; bibliographicCitation: WoRMS (2019). Janetogalathea Baba & Wicksten, 1997. Accessed at: http://www.marinespecies.org/aphia.php?p=taxdetails&id=387304 on 2019-08-23; institutionCode: CDF; collectionCode: Arthropoda; datasetName: Video transect framegrabs; basisOfRecord: HumanObservation

##### Notes

**Figure 9.**
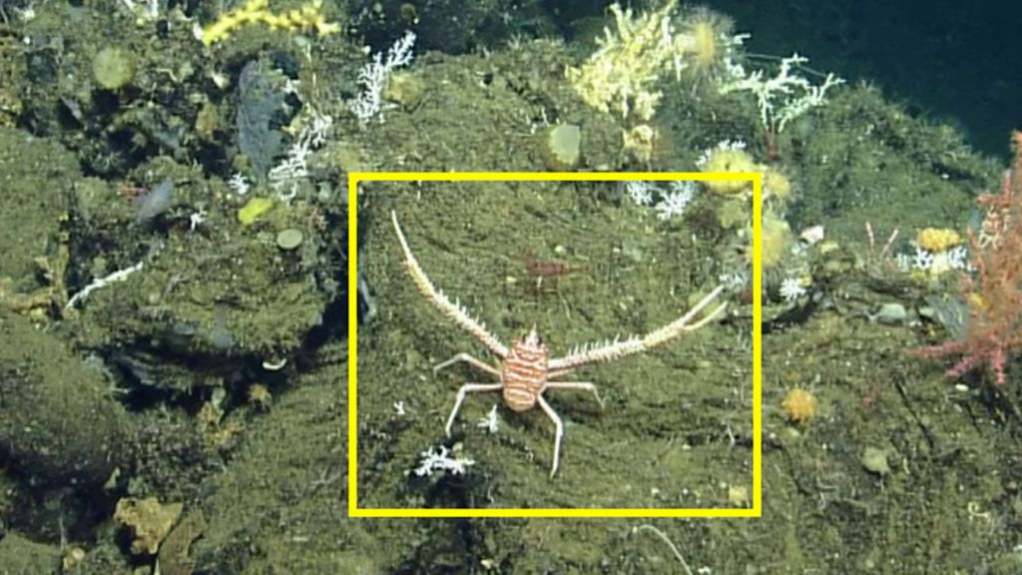
*Janetogalathea californiensis* sp. inc.

This crustacean looks much like *J. californiensis* (Baba and Wicksten 1997). If so, it constitutes a major range extension, because previous records of the species are no further south than the Gulf of California, Mexico. To date, there is only one species known of *Janetogalathea*, but the animal in the photograph differs from those previously described, because of the slender fingers of the chelae, without spines. This might be a variant of *J. californiensis* or something undescribed. Fig. 9

#### Munida

Leach, 1820

3420E85A-D675-5DE7-8A00-646DA0B16FE2

##### Materials

- **Type status:** Other material. **Occurrence:** recordedBy: CDF Volunteer; behavior: on seafloor; occurrenceStatus: present; preparations: Image only; associatedMedia: https://farm2.staticflickr.com/1912/45600632152_bd316e3674_o.png; occurrenceID: H1435_002808_Munida_sp_indet_1; **Taxon:** scientificNameID: urn:lsid:marinespecies.org:taxname:106835; scientificName: Munida; kingdom: Animalia; phylum: Arthropoda; class: Malacostraca; order: Decapoda; family: Munididae; genus: Munida; scientificNameAuthorship: Leach, 1820; taxonomicStatus: accepted; **Location:** locationID: MRGID8403; waterBody: Pacific Ocean; country: Ecuador; stateProvince: Galapagos; locality: North; verbatimLocality: East of Wolf; minimumDepthInMeters: 1148; maximumDepthInMeters: 1148; decimalLatitude: 1.2141; decimalLongitude: -91.0905; geodeticDatum: WGS84; coordinateUncertaintyInMeters: 35; **Identification:** identifiedBy: Shane Ahyong; dateIdentified: 2017; identificationRemarks: ID from imagery only; identificationQualifier: *Munida* sp. indet. 1; **Event:** eventID: NA064; samplingProtocol: Remotely Operated Vehicles; eventDate: 06-27-15; eventTime: 12:28:08 AM; habitat: Seamount; **Record Level:** language: en; bibliographicCitation: WoRMS (2019). Munida Leach, 1820. Accessed at: http://www.marinespecies.org/aphia.php?p=taxdetails&id=106835 on 2019-08-23; institutionCode: CDF; collectionCode: Arthropoda; datasetName: Video transect framegrabs; basisOfRecord: HumanObservation

##### Notes

**Figure 10.**
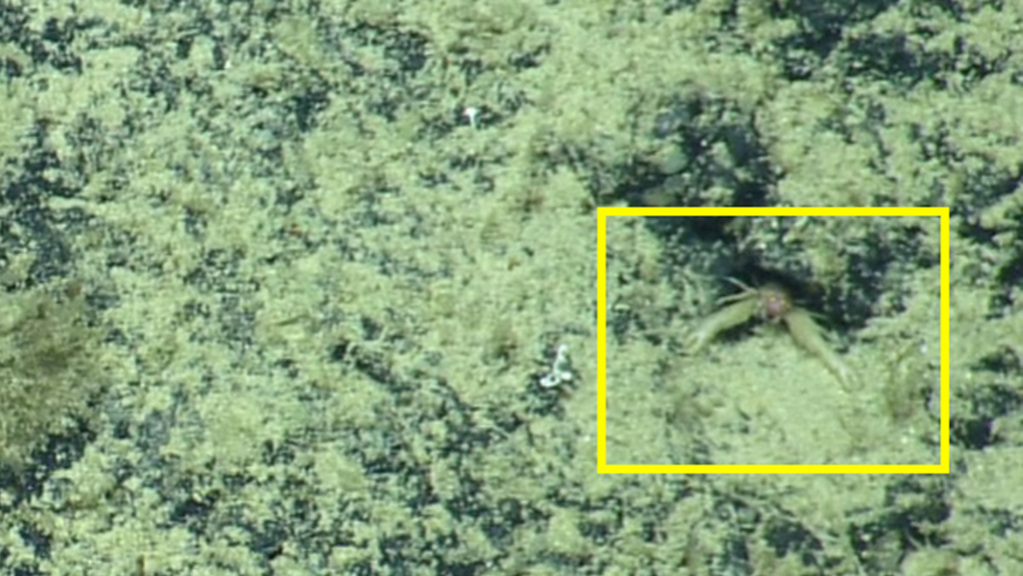
*Munida* sp. indet. 1

Appears to possess hirsute chelae. Fig. 10

#### Munida

Leach, 1820

AEEFEE11-2155-5F2F-BDEF-510805EC72C9

##### Materials

- **Type status:** Other material. **Occurrence:** recordedBy: CDF Volunteer; behavior: on seafloor; occurrenceStatus: present; preparations: Image only; associatedMedia: https://live.staticflickr.com/65535/48491312261_ef3ccf11e8_o.png; occurrenceID: H1443_175240_Munida_sp_indet_2; **Taxon:** scientificNameID: urn:lsid:marinespecies.org:taxname:106835; scientificName: Munida; kingdom: Animalia; phylum: Arthropoda; class: Malacostraca; order: Decapoda; family: Munididae; genus: Munida; scientificNameAuthorship: Leach, 1820; taxonomicStatus: accepted; **Location:** locationID: MRGID8403; waterBody: Pacific Ocean; country: Ecuador; stateProvince: Galapagos; locality: Southeast; verbatimLocality: Galapagos Platform; minimumDepthInMeters: 639; maximumDepthInMeters: 639; decimalLatitude: -0.3793; decimalLongitude: -90.811; geodeticDatum: WGS84; coordinateUncertaintyInMeters: 20; **Identification:** identifiedBy: Shane Ahyong; dateIdentified: 2017; identificationRemarks: ID from imagery only; identificationQualifier: *Munida* sp. indet. 2; **Event:** eventID: NA064; samplingProtocol: Remotely Operated Vehicles; eventDate: 07-05-15; eventTime: 5:52:40 PM; habitat: Volcanic Cone; **Record Level:** language: en; bibliographicCitation: WoRMS (2019). Munida Leach, 1820. Accessed at: http://www.marinespecies.org/aphia.php?p=taxdetails&id=106835 on 2019-08-23; institutionCode: CDF; collectionCode: Arthropoda; datasetName: Video transect framegrabs; basisOfRecord: HumanObservation

##### Notes

**Figure 11.**
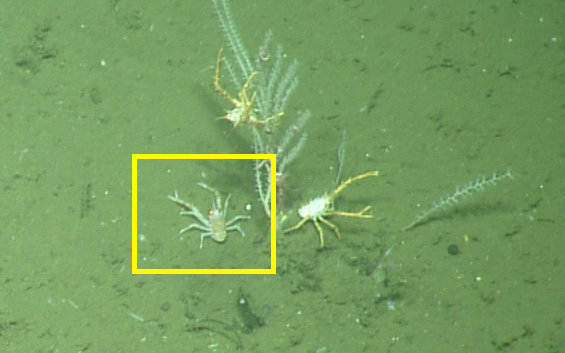
*Munida* sp. indet. 2

This species can be distinguished as different from the one shown in Fig. 9, because that one has hirsute chelae, not seen in Fig. 11.

#### Munidopsis albatrossae

Pequegnat & Pequegnat, 1973

4484DE73-28C4-557D-B9FE-64FA8F76495F

##### Materials

- **Type status:** Other material. **Occurrence:** recordedBy: CDF Volunteer; behavior: on seafloor; occurrenceStatus: present; preparations: Image only; associatedMedia: https://farm2.staticflickr.com/1958/30710222157_df94121666_o.png; occurrenceID: H1441_101331_Munidopsis_albatrossae; **Taxon:** scientificNameID: urn:lsid:marinespecies.org:taxname:378083; scientificName: Munidopsis albatrossae; kingdom: Animalia; phylum: Arthropoda; class: Malacostraca; order: Decapoda; family: Munidopsidae; genus: Munidopsis; specificEpithet: albatrossae; scientificNameAuthorship: Pequegnat & Pequegnat, 1973; taxonomicStatus: accepted; **Location:** locationID: MRGID8403; waterBody: Pacific Ocean; country: Ecuador; stateProvince: Galapagos; locality: West; verbatimLocality: West of Fernandina; minimumDepthInMeters: 3392; maximumDepthInMeters: 3392; decimalLatitude: -0.3801; decimalLongitude: -91.8972; geodeticDatum: WGS84; coordinateUncertaintyInMeters: 100; **Identification:** identifiedBy: Shane Ahyong; dateIdentified: 2017; identificationRemarks: ID from imagery only; identificationQualifier: Munidopsis albatrossae; **Event:** eventID: NA064; samplingProtocol: Remotely Operated Vehicles; eventDate: 07-03-15; eventTime: 10:13:31 AM; habitat: Lava Flow; **Record Level:** language: en; bibliographicCitation: WoRMS (2019). Munidopsis albatrossae Pequegnat & Pequegnat, 1973. Accessed at: http://www.marinespecies.org/aphia.php?p=taxdetails&id=378083 on 2019-08-23; institutionCode: CDF; collectionCode: Arthropoda; datasetName: Video transect framegrabs; basisOfRecord: HumanObservation

##### Notes

**Figure 12.**
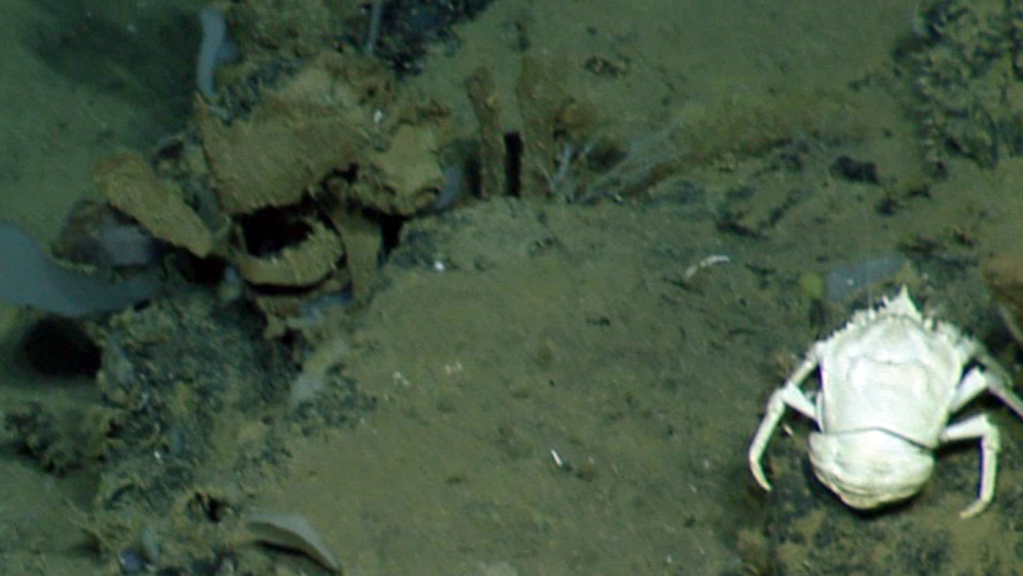
*Munidopsis albatrossae*

No further observations or comments. Fig. 12

#### Munidopsis hystrix

Faxon, 1893

BD5DCA44-174F-53F6-90D0-38E2CAC002EA

##### Materials

- **Type status:** Other material. **Occurrence:** recordedBy: CDF Volunteer; behavior: associated with octocorallia; occurrenceStatus: present; preparations: Image only; associatedMedia: https://farm2.staticflickr.com/1921/44926034384_1718f9d80d_o.png; occurrenceID: H1443_175708_Munidopsis_hystrix; **Taxon:** scientificNameID: urn:lsid:marinespecies.org:taxname:392539; scientificName: Munidopsis hystrix; kingdom: Animalia; phylum: Arthropoda; class: Malacostraca; order: Decapoda; family: Munidopsidae; genus: Munidopsis; specificEpithet: hystrix; scientificNameAuthorship: Faxon, 1893; taxonomicStatus: accepted; **Location:** locationID: MRGID8403; waterBody: Pacific Ocean; country: Ecuador; stateProvince: Galapagos; locality: Southeast; verbatimLocality: Galapagos Platform; minimumDepthInMeters: 631; maximumDepthInMeters: 631; decimalLatitude: -0.3793; decimalLongitude: -90.811; geodeticDatum: WGS84; coordinateUncertaintyInMeters: 20; **Identification:** identifiedBy: Shane Ahyong; dateIdentified: 2017; identificationRemarks: ID from imagery only; identificationQualifier: Munidopsis hystrix; **Event:** eventID: NA064; samplingProtocol: Remotely Operated Vehicles; eventDate: 07-05-15; eventTime: 5:57:08 PM; habitat: Volcanic Cone; **Record Level:** language: en; bibliographicCitation: WoRMS (2019). Munidopsis hystrix Faxon, 1893. Accessed at: http://www.marinespecies.org/aphia.php?p=taxdetails&id=392539 on 2019-08-23; institutionCode: CDF; collectionCode: Arthropoda; datasetName: Video transect framegrabs; basisOfRecord: HumanObservation
- **Type status:** Other material. **Occurrence:** recordedBy: CDF Volunteer; behavior: associated with octocorallia; occurrenceStatus: present; preparations: Image only; associatedMedia: https://farm2.staticflickr.com/1975/30710170107_0f0263a1bd_o.png; occurrenceID: H1443_175240_Munidopsis_hystrix; **Taxon:** scientificNameID: urn:lsid:marinespecies.org:taxname:392539; scientificName: Munidopsis hystrix; kingdom: Animalia; phylum: Arthropoda; class: Malacostraca; order: Decapoda; family: Munidopsidae; genus: Munidopsis; specificEpithet: hystrix; scientificNameAuthorship: Faxon, 1893; taxonomicStatus: accepted; **Location:** locationID: MRGID8403; waterBody: Pacific Ocean; country: Ecuador; stateProvince: Galapagos; locality: Southeast; verbatimLocality: Galapagos Platform; minimumDepthInMeters: 639; maximumDepthInMeters: 639; decimalLatitude: -0.3793; decimalLongitude: -90.811; geodeticDatum: WGS84; coordinateUncertaintyInMeters: 20; **Identification:** identifiedBy: Shane Ahyong; dateIdentified: 2017; identificationRemarks: ID from imagery only; identificationQualifier: Munidopsis hystrix; **Event:** eventID: NA064; samplingProtocol: Remotely Operated Vehicles; eventDate: 07-05-15; eventTime: 5:52:40 PM; habitat: Volcanic Cone; **Record Level:** language: en; bibliographicCitation: WoRMS (2019). Munidopsis hystrix Faxon, 1893. Accessed at: http://www.marinespecies.org/aphia.php?p=taxdetails&id=392539 on 2019-08-23; institutionCode: CDF; collectionCode: Arthropoda; datasetName: Video transect framegrabs; basisOfRecord: HumanObservation

##### Notes

No further observations or comments. Fig. 13

#### Munidopsis mina

Benedict, 1902

918B735C-D12F-5BCA-81BC-C6EE1ED4EC49

##### Materials

- **Type status:** Other material. **Occurrence:** recordedBy: CDF Volunteer; behavior: associated with porifera; occurrenceStatus: present; preparations: Image only; associatedMedia: https://farm2.staticflickr.com/1939/43833004130_f68f757688_o.png; occurrenceID: H1435_055907_Munidopsis_mina; **Taxon:** scientificNameID: urn:lsid:marinespecies.org:taxname:392562; scientificName: Munidopsis mina; kingdom: Animalia; phylum: Arthropoda; class: Malacostraca; order: Decapoda; family: Munidopsidae; genus: Munidopsis; specificEpithet: mina; scientificNameAuthorship: Benedict, 1902; taxonomicStatus: accepted; **Location:** locationID: MRGID8403; waterBody: Pacific Ocean; country: Ecuador; stateProvince: Galapagos; locality: North; verbatimLocality: East of Wolf; minimumDepthInMeters: 871; maximumDepthInMeters: 871; decimalLatitude: 1.2222; decimalLongitude: -91.1007; geodeticDatum: WGS84; coordinateUncertaintyInMeters: 25; **Identification:** identifiedBy: Shane Ahyong; dateIdentified: 2017; identificationRemarks: ID from imagery only; identificationQualifier: Munidopsis mina; **Event:** eventID: NA064; samplingProtocol: Remotely Operated Vehicles; eventDate: 06-27-15; eventTime: 5:59:07 AM; habitat: Seamount; **Record Level:** language: en; bibliographicCitation: WoRMS (2019). Munidopsis mina Benedict, 1902. Accessed at: http://www.marinespecies.org/aphia.php?p=taxdetails&id=392562 on 2019-08-23; institutionCode: CDF; collectionCode: Arthropoda; datasetName: Video transect framegrabs; basisOfRecord: HumanObservation

##### Notes

**Figure 14.**
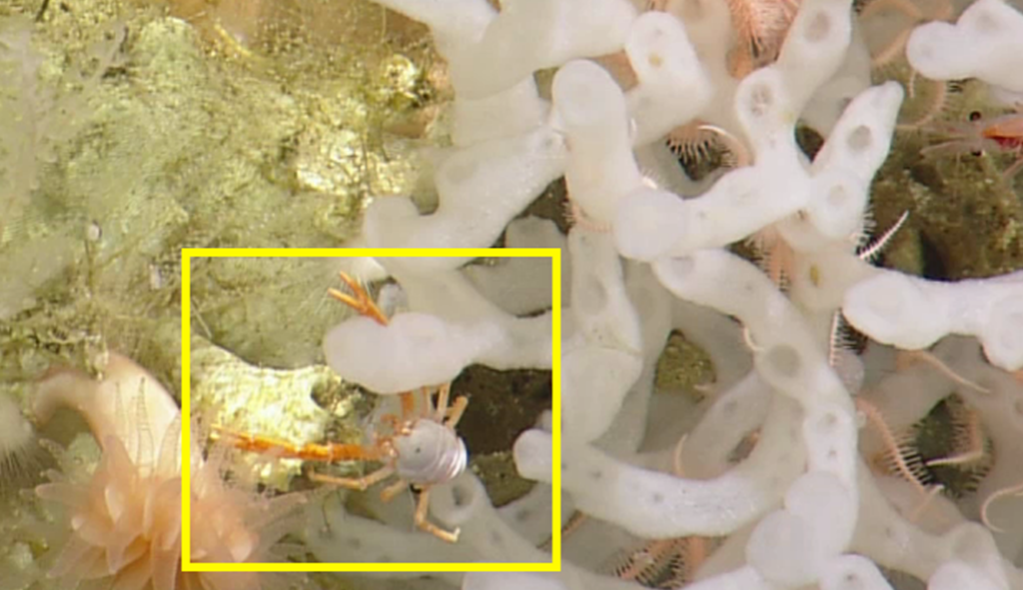
*Munidopsis mina*

No further observations or comments. Fig. 14

#### Munidopsis

Whiteaves, 1874

D4611A58-A032-5423-A239-9957AC0E4FE2

##### Materials

- **Type status:** Other material. **Occurrence:** recordedBy: CDF Volunteer; behavior: on seafloor; occurrenceStatus: present; preparations: Image only; associatedMedia: https://farm2.staticflickr.com/1957/30710220427_3a57d44d62_o.png; occurrenceID: H1435_082743_Munidopsis_sp_indet_1; **Taxon:** scientificNameID: urn:lsid:marinespecies.org:taxname:106836; scientificName: Munidopsis; kingdom: Animalia; phylum: Arthropoda; class: Malacostraca; order: Decapoda; family: Munidopsidae; genus: Munidopsis; scientificNameAuthorship: Whiteaves, 1874; taxonomicStatus: accepted; **Location:** locationID: MRGID8403; waterBody: Pacific Ocean; country: Ecuador; stateProvince: Galapagos; locality: North; verbatimLocality: East of Wolf; minimumDepthInMeters: 640; maximumDepthInMeters: 640; decimalLatitude: 1.2265; decimalLongitude: -91.1076; geodeticDatum: WGS84; coordinateUncertaintyInMeters: 20; **Identification:** identifiedBy: Shane Ahyong; dateIdentified: 2017; identificationRemarks: ID from imagery only; identificationQualifier: *Munidopsis* sp. indet. 1; **Event:** eventID: NA064; samplingProtocol: Remotely Operated Vehicles; eventDate: 06-27-15; eventTime: 8:27:43 AM; habitat: Seamount; **Record Level:** language: en; bibliographicCitation: WoRMS (2019). Munidopsis Whiteaves, 1874. Accessed at: http://www.marinespecies.org/aphia.php?p=taxdetails&id=106836 on 2019-08-23; institutionCode: CDF; collectionCode: Arthropoda; datasetName: Video transect framegrabs; basisOfRecord: HumanObservation

##### Notes

**Figure 15.**
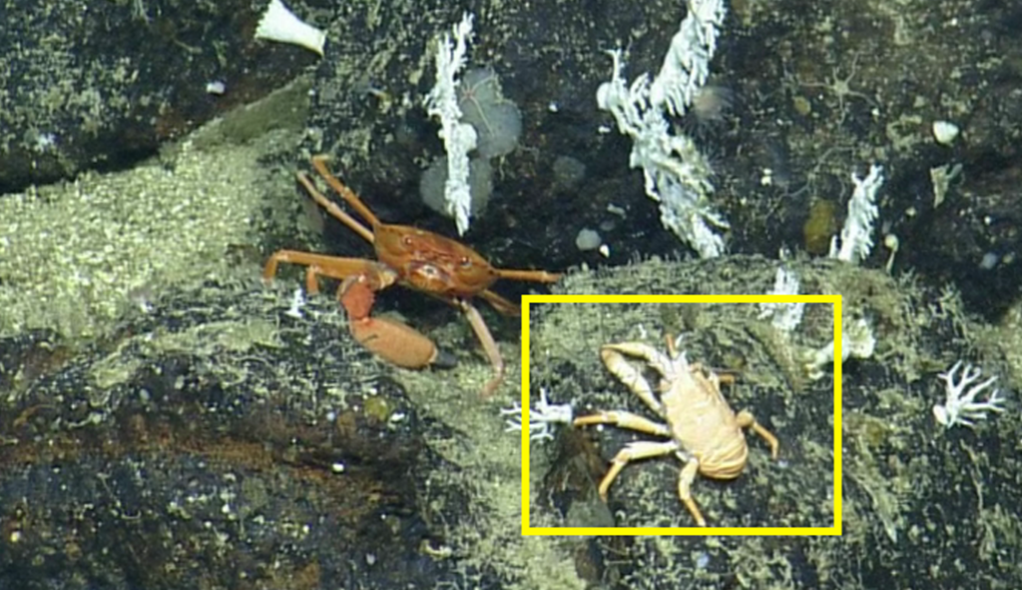
*Munidopsis* sp. indet. 1

**Figure 16.**
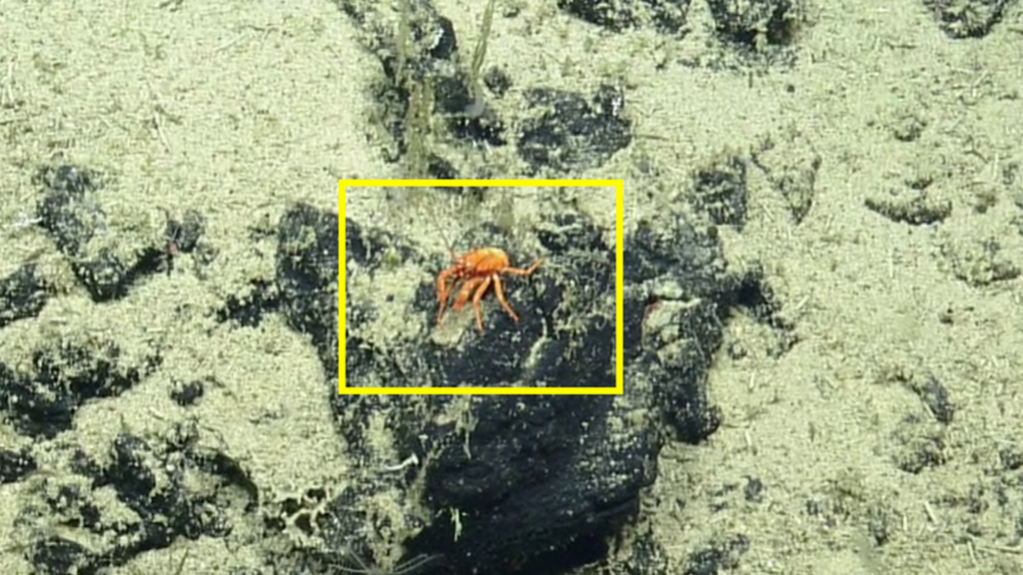
*Munidopsis* sp. indet. 2

**Figure 17.**
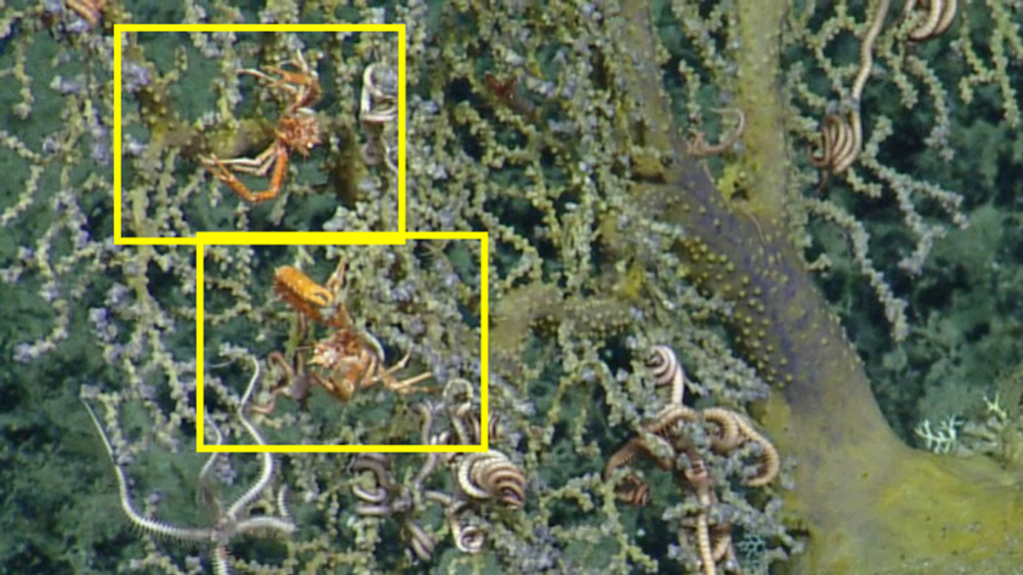
*Munidopsis* sp. indet. 3

**Figure 18.**
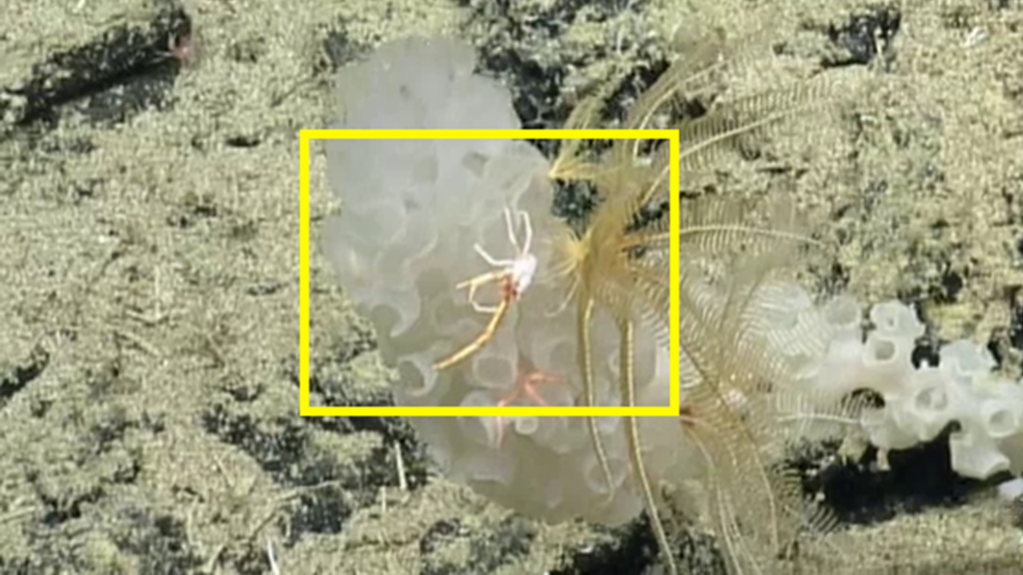
*Munidopsis* sp. indet. 4

*Munidopsis* sp. indet. 1 (Fig. 15), *Munidopsis* sp. indet. 2 (Fig. 16), *Munidopsis* sp. indet. 3 (Fig. 17) and *Munidopsis* sp. indet. 4 (Fig. 18). These images were taken at too great a distance to see fine details of the antennae, spines of the anterior carapace or other distinguishing features. However, all can be determined to belong to different species by the shape of the chelae, the colour and the length of the pereopods. Fig. 15

#### Munidopsis

Whiteaves, 1874

F6D81048-7C41-5F74-9FDF-CC166A4B55A6

##### Materials

- **Type status:** Other material. **Occurrence:** recordedBy: CDF Volunteer; behavior: on seafloor; occurrenceStatus: present; preparations: Image only; associatedMedia: https://farm2.staticflickr.com/1960/44736656285_64523ed732_o.png; occurrenceID: H1436_061913_Munidopsis_sp_indet_2; **Taxon:** scientificNameID: urn:lsid:marinespecies.org:taxname:106836; scientificName: Munidopsis; kingdom: Animalia; phylum: Arthropoda; class: Malacostraca; order: Decapoda; family: Munidopsidae; genus: Munidopsis; scientificNameAuthorship: Whiteaves, 1874; taxonomicStatus: accepted; **Location:** locationID: MRGID8403; waterBody: Pacific Ocean; country: Ecuador; stateProvince: Galapagos; locality: Far North; verbatimLocality: East of Darwin; minimumDepthInMeters: 1280; maximumDepthInMeters: 1280; decimalLatitude: 1.672; decimalLongitude: -91.6846; geodeticDatum: WGS84; coordinateUncertaintyInMeters: 40; **Identification:** identifiedBy: Shane Ahyong; dateIdentified: 2017; identificationRemarks: ID from imagery only; identificationQualifier: *Munidopsis* sp. indet. 2; **Event:** eventID: NA064; samplingProtocol: Remotely Operated Vehicles; eventDate: 06-28-15; eventTime: 6:19:13 AM; habitat: Seamount; **Record Level:** language: en; bibliographicCitation: WoRMS (2019). Munidopsis Whiteaves, 1874. Accessed at: http://www.marinespecies.org/aphia.php?p=taxdetails&id=106836 on 2019-08-23; institutionCode: CDF; collectionCode: Arthropoda; datasetName: Video transect framegrabs; basisOfRecord: HumanObservation

##### Notes

*Munidopsis* sp. indet. 1 (Fig. 15), *Munidopsis* sp. indet. 2 (Fig. 16), *Munidopsis* sp. indet. 3 (Fig. 17) and *Munidopsis* sp. indet. 4 (Fig. 18). These images were taken at too great a distance to see fine details of the antennae, spines of the anterior carapace or other distinguishing features. However, all can be determined to belong to different species by the shape of the chelae, the colour and the length of the pereopods. Fig. 16

#### Munidopsis

Whiteaves, 1874

F2F898C5-1B08-50F1-8EEE-0AD3649408AA

##### Materials

- **Type status:** Other material. **Occurrence:** recordedBy: CDF Volunteer; behavior: associated with octocorallia; occurrenceStatus: present; preparations: Image only; associatedMedia: https://farm2.staticflickr.com/1901/30710211497_5a59874c5c_o.png; occurrenceID: H1436_065826_Munidopsis_sp_indet_3; **Taxon:** scientificNameID: urn:lsid:marinespecies.org:taxname:106836; scientificName: Munidopsis; kingdom: Animalia; phylum: Arthropoda; class: Malacostraca; order: Decapoda; family: Munidopsidae; genus: Munidopsis; scientificNameAuthorship: Whiteaves, 1874; taxonomicStatus: accepted; **Location:** locationID: MRGID8403; waterBody: Pacific Ocean; country: Ecuador; stateProvince: Galapagos; locality: Far North; verbatimLocality: East of Darwin; minimumDepthInMeters: 1269; maximumDepthInMeters: 1269; decimalLatitude: 1.672; decimalLongitude: -91.6847; geodeticDatum: WGS84; coordinateUncertaintyInMeters: 40; **Identification:** identifiedBy: Shane Ahyong; dateIdentified: 2017; identificationRemarks: ID from imagery only; identificationQualifier: *Munidopsis* sp. indet. 3; **Event:** eventID: NA064; samplingProtocol: Remotely Operated Vehicles; eventDate: 06-28-15; eventTime: 6:58:26 AM; habitat: Seamount; **Record Level:** language: en; bibliographicCitation: WoRMS (2019). Munidopsis Whiteaves, 1874. Accessed at: http://www.marinespecies.org/aphia.php?p=taxdetails&id=106836 on 2019-08-23; institutionCode: CDF; collectionCode: Arthropoda; datasetName: Video transect framegrabs; basisOfRecord: HumanObservation

##### Notes

*Munidopsis* sp. indet. 1 (Fig. 15), *Munidopsis* sp. indet. 2 (Fig. 16), *Munidopsis* sp. indet. 3 (Fig. 17) and *Munidopsis* sp. indet. 4 (Fig. 18). These images were taken at too great a distance to see fine details of the antennae, spines of the anterior carapace or other distinguishing features. However, all can be determined to belong to different species by the shape of the chelae, the colour and the length of the pereopods. Fig. 17

#### Munidopsis

Whiteaves, 1874

4829E806-ACBD-5FB4-A66B-865C086638E9

##### Materials

- **Type status:** Other material. **Occurrence:** recordedBy: CDF Volunteer; behavior: associated with porifera; occurrenceStatus: present; preparations: Image only; associatedMedia: https://farm2.staticflickr.com/1938/45651075701_81007ae0bd_o.png; occurrenceID: H1436_080021_Munidopsis_sp_indet_4; **Taxon:** scientificNameID: urn:lsid:marinespecies.org:taxname:106836; scientificName: Munidopsis; kingdom: Animalia; phylum: Arthropoda; class: Malacostraca; order: Decapoda; family: Munidopsidae; genus: Munidopsis; scientificNameAuthorship: Whiteaves, 1874; taxonomicStatus: accepted; **Location:** locationID: MRGID8403; waterBody: Pacific Ocean; country: Ecuador; stateProvince: Galapagos; locality: Far North; verbatimLocality: East of Darwin; minimumDepthInMeters: 1177; maximumDepthInMeters: 1177; decimalLatitude: 1.6733; decimalLongitude: -91.6855; geodeticDatum: WGS84; coordinateUncertaintyInMeters: 35; **Identification:** identifiedBy: Shane Ahyong; dateIdentified: 2017; identificationRemarks: ID from imagery only; identificationQualifier: *Munidopsis* sp. indet. 4; **Event:** eventID: NA064; samplingProtocol: Remotely Operated Vehicles; eventDate: 06-28-15; eventTime: 8:00:21 AM; habitat: Seamount; **Record Level:** language: en; bibliographicCitation: WoRMS (2019). Munidopsis Whiteaves, 1874. Accessed at: http://www.marinespecies.org/aphia.php?p=taxdetails&id=106836 on 2019-08-23; institutionCode: CDF; collectionCode: Arthropoda; datasetName: Video transect framegrabs; basisOfRecord: HumanObservation

##### Notes

*Munidopsis* sp. indet. 1 (Fig. 15), *Munidopsis* sp. indet. 2 (Fig. 16), *Munidopsis* sp. indet. 3 (Fig. 17) and *Munidopsis* sp. indet. 4 (Fig. 18). These images were taken at too great a distance to see fine details of the antennae, spines of the anterior carapace or other distinguishing features. However, all can be determined to belong to different species by the shape of the chelae, the colour and the length of the pereopods. Fig. 18

#### Lithodoidea

Samouelle, 1819

DECED68E-48E9-56F5-8CF6-31904C9D0DA7

#### Lithodes panamensis

Faxon, 1893

B84CB056-B343-5F5B-B3BF-92F9687F9990

##### Materials

- **Type status:** Other material. **Occurrence:** recordedBy: CDF Volunteer; behavior: on seafloor; occurrenceStatus: present; preparations: Image only; associatedMedia: https://farm2.staticflickr.com/1974/44926128274_ca5a32e2a4_o.png; occurrenceID: H1435_073321_Lithodes_panamensis; **Taxon:** scientificNameID: urn:lsid:marinespecies.org:taxname:550632; scientificName: Lithodes panamensis; kingdom: Animalia; phylum: Arthropoda; class: Malacostraca; order: Decapoda; family: Lithodidae; genus: Lithodes; specificEpithet: panamensis; scientificNameAuthorship: Faxon, 1893; taxonomicStatus: accepted; **Location:** locationID: MRGID8403; waterBody: Pacific Ocean; country: Ecuador; stateProvince: Galapagos; locality: North; verbatimLocality: East of Wolf; minimumDepthInMeters: 788; maximumDepthInMeters: 788; decimalLatitude: 1.2251; decimalLongitude: -91.1036; geodeticDatum: WGS84; coordinateUncertaintyInMeters: 25; **Identification:** identifiedBy: Shane Ahyong; dateIdentified: 2017; identificationRemarks: ID from imagery only; identificationQualifier: Lithodes panamensis; **Event:** eventID: NA064; samplingProtocol: Remotely Operated Vehicles; eventDate: 06-27-15; eventTime: 7:33:21 AM; habitat: Seamount; **Record Level:** language: en; bibliographicCitation: WoRMS (2019). Lithodes panamensis Faxon, 1893. Accessed at: http://www.marinespecies.org/aphia.php?p=taxdetails&id=550632 on 2019-08-23; institutionCode: CDF; collectionCode: Arthropoda; datasetName: Video transect framegrabs; basisOfRecord: HumanObservation

##### Notes

**Figure 19.**
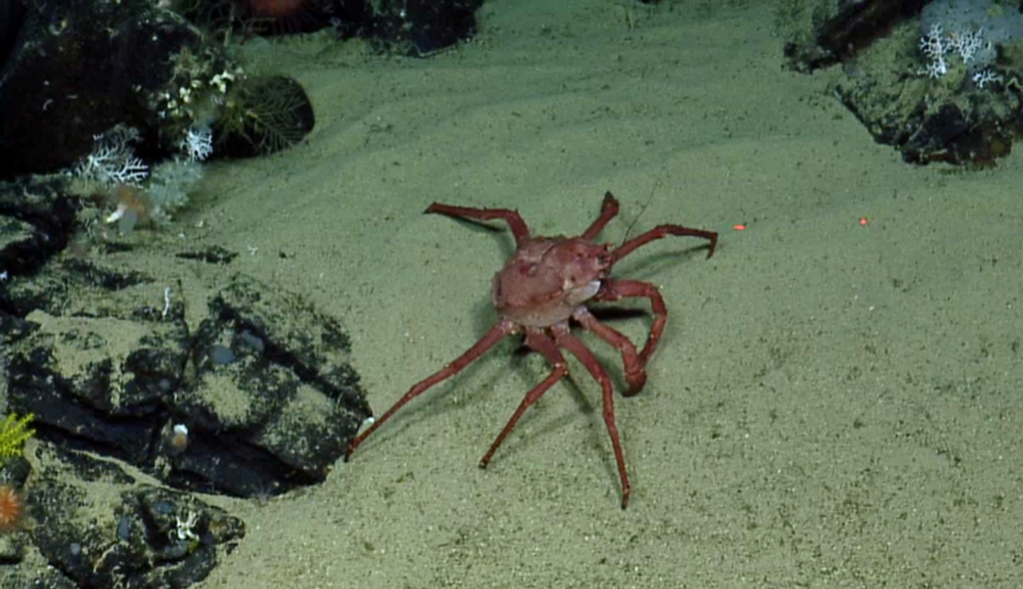
*Lithodes panamensis*

No further observations or comments. Fig. 19

#### Paralomis

White, 1856

D971BF83-A2EC-5C8E-88B5-FB06A3475BB5

##### Materials

- **Type status:** Other material. **Occurrence:** recordedBy: CDF Volunteer; behavior: on seafloor; occurrenceStatus: present; preparations: Image only; associatedMedia: https://farm2.staticflickr.com/1962/44926125524_e1902e449a_o.png; occurrenceID: H1443_210859_Paralomis_sp_indet.; **Taxon:** scientificNameID: urn:lsid:marinespecies.org:taxname:106848; scientificName: Paralomis; kingdom: Animalia; phylum: Arthropoda; class: Malacostraca; order: Decapoda; family: Lithodidae; genus: Paralomis; scientificNameAuthorship: White, 1856; taxonomicStatus: accepted; **Location:** locationID: MRGID8403; waterBody: Pacific Ocean; country: Ecuador; stateProvince: Galapagos; locality: Southeast; verbatimLocality: Galapagos Platform; minimumDepthInMeters: 441; maximumDepthInMeters: 441; decimalLatitude: -0.3727; decimalLongitude: -90.8161; geodeticDatum: WGS84; coordinateUncertaintyInMeters: 15; **Identification:** identifiedBy: Shane Ahyong; dateIdentified: 2017; identificationRemarks: ID from imagery only; identificationQualifier: *Paralomis* sp. indet.; **Event:** eventID: NA064; samplingProtocol: Remotely Operated Vehicles; eventDate: 07-05-15; eventTime: 9:08:59 PM; habitat: Volcanic Cone; **Record Level:** language: en; bibliographicCitation: WoRMS (2019). Paralomis White, 1856. Accessed at: http://www.marinespecies.org/aphia.php?p=taxdetails&id=106848 on 2019-08-23; institutionCode: CDF; collectionCode: Arthropoda; datasetName: Video transect framegrabs; basisOfRecord: HumanObservation

##### Notes

**Figure 20.**
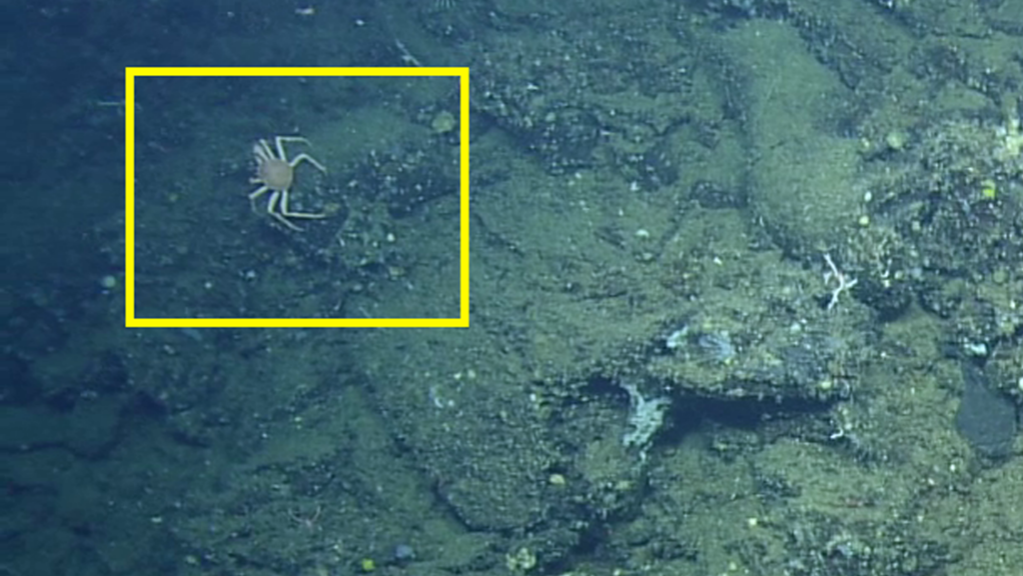
*Paralomis* sp. indet.

No further observations or comments. Fig. 20

#### Paguroidea

Latreille, 1802

4D990E54-BDB7-5217-B905-A22BE2A71BC5

#### Probeebei mirabilis

Boone, 1926

0BE0037D-3D30-5CA4-932D-D9DA9CA54908

##### Materials

- **Type status:** Other material. **Occurrence:** recordedBy: CDF Volunteer; behavior: on seafloor; occurrenceStatus: present; preparations: Image only; associatedMedia: https://farm2.staticflickr.com/1931/45651016991_4ccd4af5d8_o.png; occurrenceID: H1441_095146_Probeebei_mirabilis; **Taxon:** scientificNameID: urn:lsid:marinespecies.org:taxname:368012; scientificName: Probeebei mirabilis; kingdom: Animalia; phylum: Arthropoda; class: Malacostraca; order: Decapoda; family: Parapaguridae; genus: Probeebei; specificEpithet: mirabilis; scientificNameAuthorship: Boone, 1926; taxonomicStatus: accepted; **Location:** locationID: MRGID8403; waterBody: Pacific Ocean; country: Ecuador; stateProvince: Galapagos; locality: West; verbatimLocality: West of Fernandina; minimumDepthInMeters: 3393; maximumDepthInMeters: 3393; decimalLatitude: -0.3789; decimalLongitude: -91.8991; geodeticDatum: WGS84; coordinateUncertaintyInMeters: 100; **Identification:** identifiedBy: Shane Ahyong; dateIdentified: 2017; identificationRemarks: ID from imagery only; identificationQualifier: Probeebei mirabilis; **Event:** eventID: NA064; samplingProtocol: Remotely Operated Vehicles; eventDate: 07-03-15; eventTime: 9:51:46 AM; habitat: Lava Flow; **Record Level:** language: en; bibliographicCitation: Lemaitre, R.; McLaughlin, P. (2019). World Paguroidea & Lomisoidea database. Probeebei mirabilis Boone, 1926. Accessed through: World Register of Marine Species at: http://www.marinespecies.org/aphia.php?p=taxdetails&id=368012 on 2019-08-23; institutionCode: CDF; collectionCode: Arthropoda; datasetName: Video transect framegrabs; basisOfRecord: HumanObservation

##### Notes

**Figure 21.**
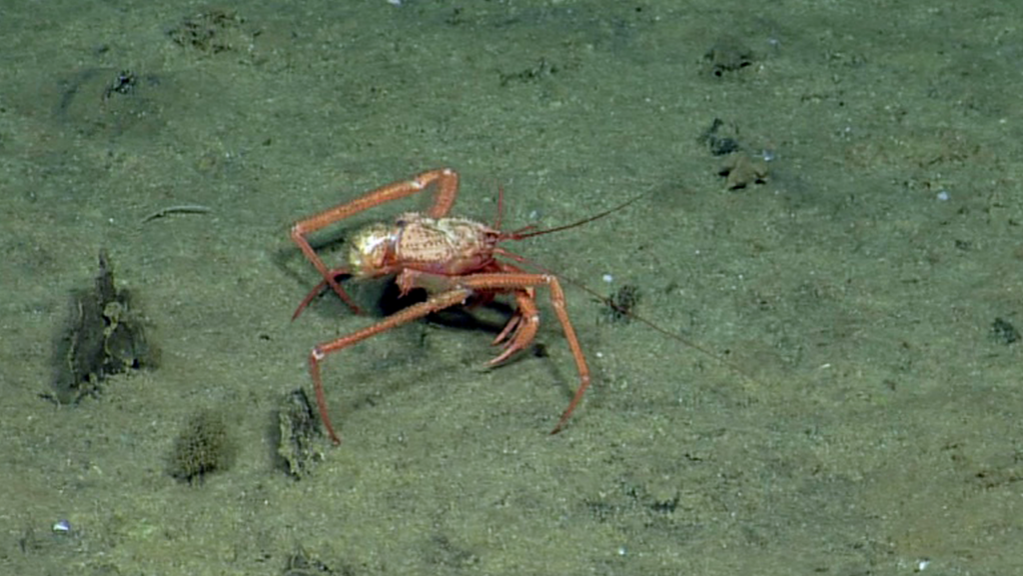
*Probeebei mirabilis*

No further observations or comments. Fig. 21

#### Tylaspis anomala

Henderson, 1885

562F2D8F-E7B3-589E-9F33-8B6B5396EAE2

##### Materials

- **Type status:** Other material. **Occurrence:** recordedBy: CDF Volunteer; behavior: on seafloor; occurrenceStatus: present; preparations: Image only; associatedMedia: https://farm2.staticflickr.com/1921/45651006891_6c828df1f0_o.png; occurrenceID: H1436_012101_Tylaspis_anomala; **Taxon:** scientificNameID: urn:lsid:marinespecies.org:taxname:368016; scientificName: Tylaspis anomala; kingdom: Animalia; phylum: Arthropoda; class: Malacostraca; order: Decapoda; family: Parapaguridae; genus: Tylaspis; specificEpithet: anomala; scientificNameAuthorship: Henderson, 1885; taxonomicStatus: accepted; **Location:** locationID: MRGID8403; waterBody: Pacific Ocean; country: Ecuador; stateProvince: Galapagos; locality: Far North; verbatimLocality: East of Darwin; minimumDepthInMeters: 2075; maximumDepthInMeters: 2075; decimalLatitude: 1.6592; decimalLongitude: -91.6739; geodeticDatum: WGS84; coordinateUncertaintyInMeters: 60; **Identification:** identifiedBy: Shane Ahyong; dateIdentified: 2017; identificationRemarks: ID from imagery only; identificationQualifier: Tylaspis anomala; **Event:** eventID: NA064; samplingProtocol: Remotely Operated Vehicles; eventDate: 06-28-15; eventTime: 1:21:01 AM; habitat: Seamount; **Record Level:** language: en; bibliographicCitation: Lemaitre, R.; McLaughlin, P. (2019). World Paguroidea & Lomisoidea database. Tylaspis anomala Henderson, 1885. Accessed through: World Register of Marine Species at: http://www.marinespecies.org/aphia.php?p=taxdetails&id=368016 on 2019-08-23; institutionCode: CDF; collectionCode: Arthropoda; datasetName: Video transect framegrabs; basisOfRecord: HumanObservation

##### Notes

**Figure 22.**
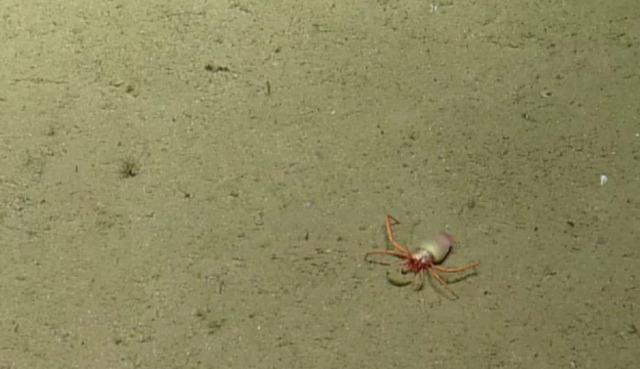
*Tylaspis anomala*

This observation is a new record for Galapagos. Notice that a sea anemone has overgrown the abdomen of the crab, a common association found in this family of hermit crabs. Fig. 22

#### Brachyura

Linnaeus‎, ‎1758

44D7A0B3-C8A3-5E35-A7F1-4FE5DAB0E7B0

#### Cancroidea

Latreille, 1802

BCFB322C-4656-52A0-97D8-3CFC882731BD

#### Metacarcinus

A. Milne-Edwards, 1862

47BC0C44-BC27-5D4F-B0DE-9C3CC8A17F3D

##### Materials

- **Type status:** Other material. **Occurrence:** recordedBy: CDF Volunteer; behavior: on seafloor; occurrenceStatus: present; preparations: Image only; associatedMedia: https://farm2.staticflickr.com/1913/31778740868_c9cf226f55_o.png; occurrenceID: H1435_122925_Metacarcinus_sp_inc_edwardsii; **Taxon:** scientificNameID: urn:lsid:marinespecies.org:taxname:439213; scientificName: Metacarcinus; kingdom: Animalia; phylum: Arthropoda; class: Malacostraca; order: Decapoda; family: Cancridae; genus: Metacarcinus; scientificNameAuthorship: A. Milne-Edwards, 1862; taxonomicStatus: accepted; **Location:** locationID: MRGID8403; waterBody: Pacific Ocean; country: Ecuador; stateProvince: Galapagos; locality: North; verbatimLocality: East of Wolf; minimumDepthInMeters: 327; maximumDepthInMeters: 327; decimalLatitude: 1.2319; decimalLongitude: -91.1137; geodeticDatum: WGS84; coordinateUncertaintyInMeters: 10; **Identification:** identifiedBy: Shane Ahyong; dateIdentified: 2017; identificationRemarks: ID from imagery only; identificationQualifier: *Metacarcinus edwardsii* sp. inc.; **Event:** eventID: NA064; samplingProtocol: Remotely Operated Vehicles; eventDate: 06-27-15; eventTime: 12:29:25 PM; habitat: Seamount; **Record Level:** language: en; bibliographicCitation: WoRMS (2019). Metacarcinus A. Milne-Edwards, 1862. Accessed at: http://www.marinespecies.org/aphia.php?p=taxdetails&id=439213 on 2019-08-23; institutionCode: CDF; collectionCode: Arthropoda; datasetName: Video transect framegrabs; basisOfRecord: HumanObservation

##### Notes

**Figure 23.**
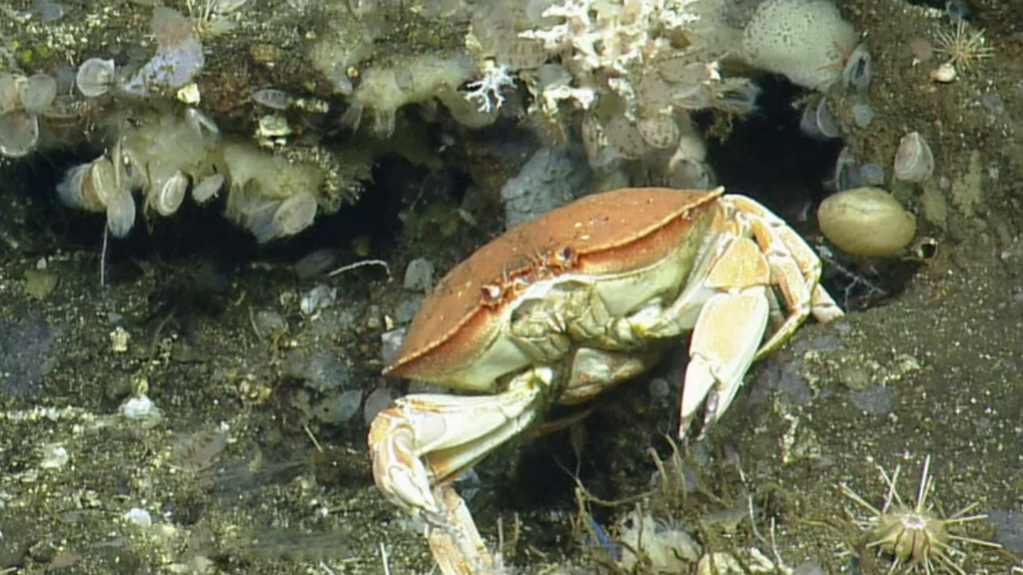
*Metacarcinus edwardsii* sp. inc.

It is very clear from the form of the carapace and chelipeds that this is a member of the Cancridae and subtle details of the carapace margins visible in other photos correspond best to *Metacarcinus*. Fig. 23

#### Goneplacoidea

MacLeay, 1838

78CEFA11-55AE-50B5-86DA-5F7526D0FD3D

#### Mathildellidae

Karasawa & Kato, 2003

C2DB0731-7E85-56D8-AFA1-516AFA5C2ADC

##### Materials

- **Type status:** Other material. **Occurrence:** recordedBy: CDF Volunteer; behavior: on seafloor; occurrenceStatus: present; preparations: Image only; associatedMedia: https://farm2.staticflickr.com/1976/31778722608_435173cc58_o.png; occurrenceID: H1435_082740_Mathildellidae_gen_indet.; **Taxon:** scientificNameID: urn:lsid:marinespecies.org:taxname:439044; scientificName: Mathildellidae; kingdom: Animalia; phylum: Arthropoda; class: Malacostraca; order: Decapoda; family: Mathildellidae; scientificNameAuthorship: Karasawa & Kato, 2003; taxonomicStatus: accepted; **Location:** locationID: MRGID8403; waterBody: Pacific Ocean; country: Ecuador; stateProvince: Galapagos; locality: North; verbatimLocality: East of Wolf; minimumDepthInMeters: 640; maximumDepthInMeters: 640; decimalLatitude: 1.2261; decimalLongitude: -91.1076; geodeticDatum: WGS84; coordinateUncertaintyInMeters: 15; **Identification:** identifiedBy: Shane Ahyong; dateIdentified: 2017; identificationRemarks: ID from imagery only; identificationQualifier: Mathildellidae gen. indet.; **Event:** eventID: NA064; samplingProtocol: Remotely Operated Vehicles; eventDate: 06-27-15; eventTime: 8:27:40 AM; habitat: Seamount; **Record Level:** language: en; bibliographicCitation: WoRMS (2019). Mathildellidae Karasawa & Kato, 2003. Accessed at: http://www.marinespecies.org/aphia.php?p=taxdetails&id=439044 on 2019-08-23; institutionCode: CDF; collectionCode: Arthropoda; datasetName: Video transect framegrabs; basisOfRecord: HumanObservation
- **Type status:** Other material. **Occurrence:** recordedBy: CDF Volunteer; behavior: on seafloor; occurrenceStatus: present; preparations: Image only; associatedMedia: https://farm2.staticflickr.com/1962/44926123184_df7f396d15_o.png; occurrenceID: H1435_085954_Mathildellidae_gen_indet.; **Taxon:** scientificNameID: urn:lsid:marinespecies.org:taxname:439044; scientificName: Mathildellidae; kingdom: Animalia; phylum: Arthropoda; class: Malacostraca; order: Decapoda; family: Mathildellidae; scientificNameAuthorship: Karasawa & Kato, 2003; taxonomicStatus: accepted; **Location:** locationID: MRGID8403; waterBody: Pacific Ocean; country: Ecuador; stateProvince: Galapagos; locality: North; verbatimLocality: East of Wolf; minimumDepthInMeters: 512; maximumDepthInMeters: 512; decimalLatitude: 1.2271; decimalLongitude: -91.1099; geodeticDatum: WGS84; coordinateUncertaintyInMeters: 15; **Identification:** identifiedBy: Shane Ahyong; dateIdentified: 2017; identificationRemarks: ID from imagery only; identificationQualifier: Mathildellidae gen. indet.; **Event:** eventID: NA064; samplingProtocol: Remotely Operated Vehicles; eventDate: 06-27-15; eventTime: 8:59:54 AM; habitat: Seamount; **Record Level:** language: en; bibliographicCitation: WoRMS (2019). Mathildellidae Karasawa & Kato, 2003. Accessed at: http://www.marinespecies.org/aphia.php?p=taxdetails&id=439044 on 2019-08-23; institutionCode: CDF; collectionCode: Arthropoda; datasetName: Video transect framegrabs; basisOfRecord: HumanObservation

##### Notes

Appears to be a goneplacoid crab. The overall habitus, aspects of the shape and colouration of the claws and shape of the carapace all fit well with Mathildellidae, based on the author's (S.Ahyong) extensive examination of many species of this group. Fig. 24

#### Homoloidea

De Haan, 1839

ABD2F657-8197-543D-B002-F8C55DC49FED

#### Paromola

Wood-Mason in Wood-Mason & Alcock, 1891

856C7C5C-A125-5232-AC15-E2AE6C786BEF

##### Materials

- **Type status:** Other material. **Occurrence:** recordedBy: CDF Volunteer; behavior: on seafloor; occurrenceStatus: present; preparations: Image only; associatedMedia: https://farm2.staticflickr.com/1957/31778735478_806fcb3334_o.png; occurrenceID: H1435_140039_Paromola_sp_inc_rathbunae; **Taxon:** scientificNameID: urn:lsid:marinespecies.org:taxname:106869; scientificName: Paromola; kingdom: Animalia; phylum: Arthropoda; class: Malacostraca; order: Decapoda; family: Homolidae; genus: Paromola; scientificNameAuthorship: Wood-Mason in Wood-Mason & Alcock, 1891; taxonomicStatus: accepted; **Location:** locationID: MRGID8403; waterBody: Pacific Ocean; country: Ecuador; stateProvince: Galapagos; locality: North; verbatimLocality: East of Wolf; minimumDepthInMeters: 330; maximumDepthInMeters: 330; decimalLatitude: 1.2341; decimalLongitude: -91.1142; geodeticDatum: WGS84; coordinateUncertaintyInMeters: 10; **Identification:** identifiedBy: Shane Ahyong; dateIdentified: 2017; identificationRemarks: ID from imagery only; identificationQualifier: *Paromola rathbunae* sp. inc.; **Event:** eventID: NA064; samplingProtocol: Remotely Operated Vehicles; eventDate: 06-27-15; eventTime: 2:00:39 PM; habitat: Seamount; **Record Level:** language: en; bibliographicCitation: WoRMS (2019). Paromola Wood-Mason in Wood-Mason & Alcock, 1891. Accessed at: http://www.marinespecies.org/aphia.php?p=taxdetails&id=106869 on 2019-08-23; institutionCode: CDF; collectionCode: Arthropoda; datasetName: Video transect framegrabs; basisOfRecord: HumanObservation
- **Type status:** Other material. **Occurrence:** recordedBy: CDF Volunteer; behavior: on seafloor; occurrenceStatus: present; preparations: Image only; associatedMedia: https://farm2.staticflickr.com/1905/44926138884_4c0525a590_o.png; occurrenceID: H1435_105759_Paromola_sp_inc_rathbunae; **Taxon:** scientificNameID: urn:lsid:marinespecies.org:taxname:106869; scientificName: Paromola; kingdom: Animalia; phylum: Arthropoda; class: Malacostraca; order: Decapoda; family: Homolidae; genus: Paromola; scientificNameAuthorship: Wood-Mason in Wood-Mason & Alcock, 1891; taxonomicStatus: accepted; **Location:** locationID: MRGID8403; waterBody: Pacific Ocean; country: Ecuador; stateProvince: Galapagos; locality: North; verbatimLocality: East of Wolf; minimumDepthInMeters: 364; maximumDepthInMeters: 364; decimalLatitude: 1.2289; decimalLongitude: -91.1122; geodeticDatum: WGS84; coordinateUncertaintyInMeters: 10; **Identification:** identifiedBy: Shane Ahyong; dateIdentified: 2017; identificationRemarks: ID from imagery only; identificationQualifier: *Paromola rathbunae* sp. inc.; **Event:** eventID: NA064; samplingProtocol: Remotely Operated Vehicles; eventDate: 06-27-15; eventTime: 10:57:59 AM; habitat: Seamount; **Record Level:** language: en; bibliographicCitation: WoRMS (2019). Paromola Wood-Mason in Wood-Mason & Alcock, 1891. Accessed at: http://www.marinespecies.org/aphia.php?p=taxdetails&id=106869 on 2019-08-23; institutionCode: CDF; collectionCode: Arthropoda; datasetName: Video transect framegrabs; basisOfRecord: HumanObservation
- **Type status:** Other material. **Occurrence:** recordedBy: CDF Volunteer; behavior: on seafloor; occurrenceStatus: present; preparations: Image only; associatedMedia: https://farm2.staticflickr.com/1908/31778730058_6476463bd8_o.png; occurrenceID: H1435_133848_Paromola_sp_inc_rathbunae; **Taxon:** scientificNameID: urn:lsid:marinespecies.org:taxname:106869; scientificName: Paromola; kingdom: Animalia; phylum: Arthropoda; class: Malacostraca; order: Decapoda; family: Homolidae; genus: Paromola; scientificNameAuthorship: Wood-Mason in Wood-Mason & Alcock, 1891; taxonomicStatus: accepted; **Location:** locationID: MRGID8403; waterBody: Pacific Ocean; country: Ecuador; stateProvince: Galapagos; locality: North; verbatimLocality: East of Wolf; minimumDepthInMeters: 317; maximumDepthInMeters: 317; decimalLatitude: 1.2334; decimalLongitude: -91.1143; geodeticDatum: WGS84; coordinateUncertaintyInMeters: 10; **Identification:** identifiedBy: Shane Ahyong; dateIdentified: 2017; identificationRemarks: ID from imagery only; identificationQualifier: *Paromola rathbunae* sp. inc.; **Event:** eventID: NA064; samplingProtocol: Remotely Operated Vehicles; eventDate: 06-27-15; eventTime: 1:38:48 PM; habitat: Seamount; **Record Level:** language: en; bibliographicCitation: WoRMS (2019). Paromola Wood-Mason in Wood-Mason & Alcock, 1891. Accessed at: http://www.marinespecies.org/aphia.php?p=taxdetails&id=106869 on 2019-08-23; institutionCode: CDF; collectionCode: Arthropoda; datasetName: Video transect framegrabs; basisOfRecord: HumanObservation
- **Type status:** Other material. **Occurrence:** recordedBy: CDF Volunteer; behavior: on seafloor; occurrenceStatus: present; preparations: Image only; associatedMedia: https://farm2.staticflickr.com/1943/44926133174_b2fdece71d_o.png; occurrenceID: H1435_133840_Paromola_sp_inc_rathbunae; **Taxon:** scientificNameID: urn:lsid:marinespecies.org:taxname:106869; scientificName: Paromola; kingdom: Animalia; phylum: Arthropoda; class: Malacostraca; order: Decapoda; family: Homolidae; genus: Paromola; scientificNameAuthorship: Wood-Mason in Wood-Mason & Alcock, 1891; taxonomicStatus: accepted; **Location:** locationID: MRGID8403; waterBody: Pacific Ocean; country: Ecuador; stateProvince: Galapagos; locality: North; verbatimLocality: East of Wolf; minimumDepthInMeters: 316; maximumDepthInMeters: 316; decimalLatitude: 1.2334; decimalLongitude: -91.1143; geodeticDatum: WGS84; coordinateUncertaintyInMeters: 10; **Identification:** identifiedBy: Shane Ahyong; dateIdentified: 2017; identificationRemarks: ID from imagery only; identificationQualifier: *Paromola rathbunae* sp. inc.; **Event:** eventID: NA064; samplingProtocol: Remotely Operated Vehicles; eventDate: 06-27-15; eventTime: 1:38:40 PM; habitat: Seamount; **Record Level:** language: en; bibliographicCitation: WoRMS (2019). Paromola Wood-Mason in Wood-Mason & Alcock, 1891. Accessed at: http://www.marinespecies.org/aphia.php?p=taxdetails&id=106869 on 2019-08-23; institutionCode: CDF; collectionCode: Arthropoda; datasetName: Video transect framegrabs; basisOfRecord: HumanObservation

##### Notes

This observation is a new record for Galapagos. Fig. 25

#### Majoidea

Samouelle, 1819

F6FEC4F1-CB89-569D-B727-A3C521F8136C

#### Lophorochinia parabranchia

Garth, 1969

1BF4830D-421F-5C3E-B8E0-0FF79FB3BB0A

##### Materials

- **Type status:** Other material. **Occurrence:** recordedBy: CDF Volunteer; behavior: on seafloor; occurrenceStatus: present; preparations: Image only; associatedMedia: https://farm2.staticflickr.com/1954/45600657542_2583e69769_o.png; occurrenceID: H1435_091549_Lophorochinia_parabranchia; **Taxon:** scientificNameID: urn:lsid:marinespecies.org:taxname:441461; scientificName: Lophorochinia parabranchia; kingdom: Animalia; phylum: Arthropoda; class: Malacostraca; order: Decapoda; family: Epialtidae; genus: Lophorochinia; specificEpithet: parabranchia; scientificNameAuthorship: Garth, 1969; taxonomicStatus: accepted; **Location:** locationID: MRGID8403; waterBody: Pacific Ocean; country: Ecuador; stateProvince: Galapagos; locality: North; verbatimLocality: East of Wolf; minimumDepthInMeters: 445; maximumDepthInMeters: 445; decimalLatitude: 1.2274; decimalLongitude: -91.1108; geodeticDatum: WGS84; coordinateUncertaintyInMeters: 15; **Identification:** identifiedBy: Shane Ahyong; dateIdentified: 2017; identificationRemarks: ID from imagery only; identificationQualifier: Lophorochinia parabranchia; **Event:** eventID: NA064; samplingProtocol: Remotely Operated Vehicles; eventDate: 06-27-15; eventTime: 9:15:49 AM; habitat: Seamount; **Record Level:** language: en; bibliographicCitation: WoRMS (2019). Lophorochinia parabranchia Garth, 1969. Accessed at: http://www.marinespecies.org/aphia.php?p=taxdetails&id=441461 on 2019-08-23; institutionCode: CDF; collectionCode: Arthropoda; datasetName: Video transect framegrabs; basisOfRecord: HumanObservation

##### Notes

**Figure 26.**
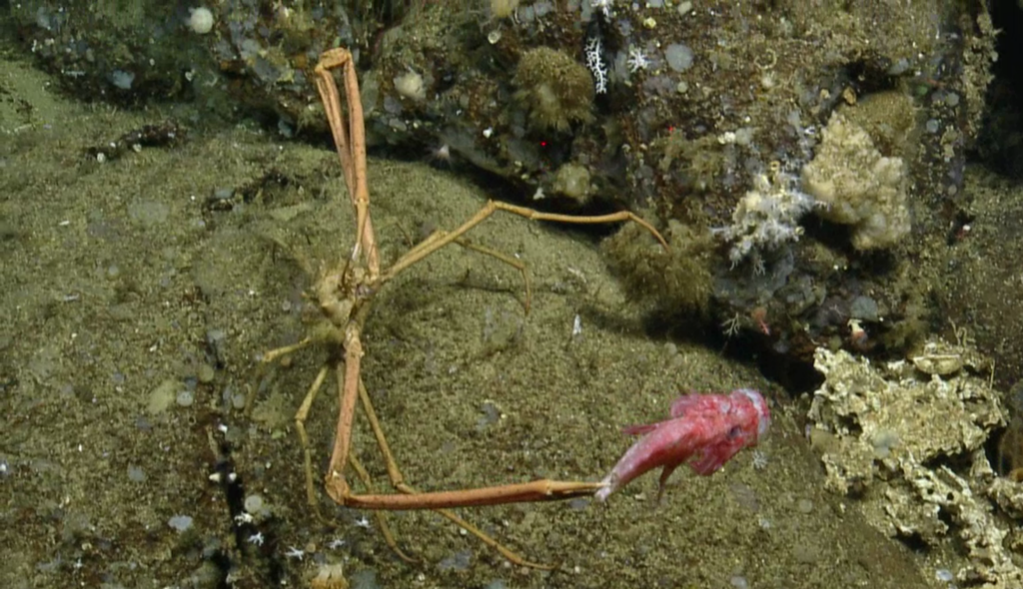
*Lophorochinia parabranchia*

No further observations or comments. Fig. 26

#### Caridea

‎Dana‎, 1852

84518000-981E-5CEA-8509-A08C17AE586B

#### Campylonotoidea

Sollaud, 1913

E768022D-878C-53CA-AFAE-56434A5202C3

#### Bathypalaemonella

Balss, 1914

4417201C-6417-5FD8-82FA-9D6219703629

##### Materials

- **Type status:** Other material. **Occurrence:** recordedBy: CDF Volunteer; behavior: associated with antipatharia; occurrenceStatus: present; preparations: Image only; associatedMedia: https://farm2.staticflickr.com/1937/45651139141_a79abd964d_o.png; occurrenceID: H1435_054749_Bathypalaemonella_sp_indet.; **Taxon:** scientificNameID: urn:lsid:marinespecies.org:taxname:107004; scientificName: Bathypalaemonella; kingdom: Animalia; phylum: Arthropoda; class: Malacostraca; order: Decapoda; family: Bathypalaemonellidae; genus: Bathypalaemonella; scientificNameAuthorship: Balss, 1914; taxonomicStatus: accepted; **Location:** locationID: MRGID8403; waterBody: Pacific Ocean; country: Ecuador; stateProvince: Galapagos; locality: North; verbatimLocality: East of Wolf; minimumDepthInMeters: 872; maximumDepthInMeters: 872; decimalLatitude: 1.2222; decimalLongitude: -91.1007; geodeticDatum: WGS84; coordinateUncertaintyInMeters: 25; **Identification:** identifiedBy: Mary Wicksten; dateIdentified: 2017; identificationRemarks: ID from imagery only; identificationQualifier: *Bathypalaemonella* sp. indet.; **Event:** eventID: NA064; samplingProtocol: Remotely Operated Vehicles; eventDate: 06-27-15; eventTime: 5:47:49 AM; habitat: Seamount; **Record Level:** language: en; bibliographicCitation: WoRMS (2019). Bathypalaemonella Balss, 1914. Accessed at: http://www.marinespecies.org/aphia.php?p=taxdetails&id=107004 on 2019-08-23; institutionCode: CDF; collectionCode: Arthropoda; datasetName: Video transect framegrabs; basisOfRecord: HumanObservation

##### Notes

**Figure 27.**
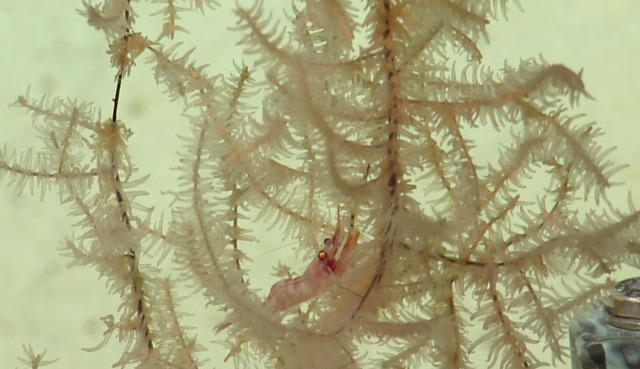
*Bathypalaemonella* sp. indet.

This could be *Bathypalaemonella delsolari* (Wicksten and Mendez 1983), the only species of this genus reported in the eastern Pacific. However, further identification would require a specimen as characteristic features of the chelipeds cannot be seen in the photograph. Fig. 27

#### Nematocarcinoidea

Smith, 1884

751EC8EA-02B5-57E1-B1CA-34079F20F9B7

#### Nematocarcinus

A. Milne-Edwards, 1881

EECD841D-53CA-57A4-BAB6-70A70DC18F5B

##### Materials

- **Type status:** Other material. **Occurrence:** recordedBy: CDF Volunteer; behavior: in water column; occurrenceStatus: present; preparations: Image | 75% ETOH; associatedMedia: https://farm2.staticflickr.com/1953/45651136511_6395079c62_o.png; occurrenceID: H1436_040940_Nematocarcinus_sp_indet.; **Taxon:** scientificNameID: urn:lsid:marinespecies.org:taxname:107015; scientificName: Nematocarcinus; kingdom: Animalia; phylum: Arthropoda; class: Malacostraca; order: Decapoda; family: Nematocarcinidae; genus: Nematocarcinus; scientificNameAuthorship: A. Milne-Edwards, 1881; taxonomicStatus: accepted; **Location:** locationID: MRGID8403; waterBody: Pacific Ocean; country: Ecuador; stateProvince: Galapagos; locality: Far North; verbatimLocality: East of Darwin; minimumDepthInMeters: 1607; maximumDepthInMeters: 1607; decimalLatitude: 1.6687; decimalLongitude: -91.6815; geodeticDatum: WGS84; coordinateUncertaintyInMeters: 50; **Identification:** identifiedBy: Mary Wicksten | Tim Shank; dateIdentified: 2017; identificationRemarks: Image ID confirmed from genetics; identificationQualifier: *Nematocarcinus* sp. indet.; **Event:** eventID: NA064; samplingProtocol: Remotely Operated Vehicles; eventDate: 06-28-15; eventTime: 4:09:40 AM; habitat: Seamount; **Record Level:** language: en; bibliographicCitation: WoRMS (2019). Nematocarcinus A. Milne-Edwards, 1881. Accessed at: http://www.marinespecies.org/aphia.php?p=taxdetails&id=107015 on 2019-08-23; institutionCode: CDF; collectionCode: Arthropoda; datasetName: Video transect framegrabs; basisOfRecord: HumanObservation

##### Notes

**Figure 28.**
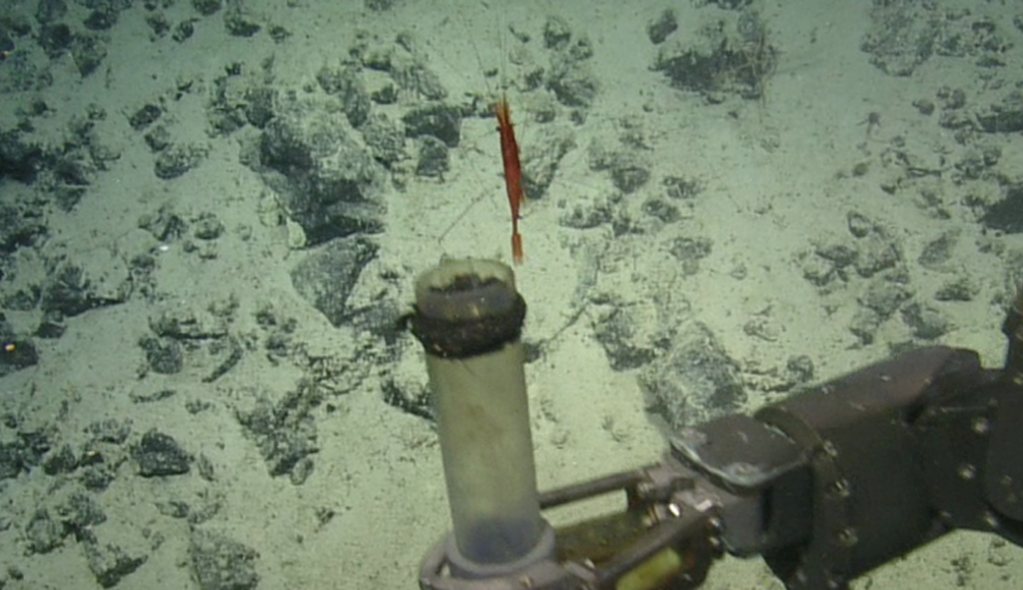
*Nematocarcinus* sp. indet.

Reported from Peru (Sample NA064-022-01-01-A). Genetics could not provide idenfication beyond genus. The images were taken too far away to see features of the teeth on the rostrum or the relative length of the rostrum to the carapace and so neither Fig. 27 nor Fig. 28 can be identified beyond *Nematocarcinus*. Furthermore, it cannot be determined whether they are the same species or not. Species of Nematocarcinus have elongate, thread-like legs, barely visible in Fig. 28.

#### Nematocarcinus

A. Milne-Edwards, 1881

861ADEEB-A5E5-58E0-BAF2-D91216C0E9BE

##### Materials

- **Type status:** Other material. **Occurrence:** recordedBy: CDF Volunteer; behavior: on seafloor; occurrenceStatus: present; preparations: Image only; associatedMedia: https://farm2.staticflickr.com/1950/45651134551_4a81276830_o.png; occurrenceID: H1441_115638_Nematocarcinus_sp_indet.; **Taxon:** scientificNameID: urn:lsid:marinespecies.org:taxname:107015; scientificName: Nematocarcinus; kingdom: Animalia; phylum: Arthropoda; class: Malacostraca; order: Decapoda; family: Nematocarcinidae; genus: Nematocarcinus; scientificNameAuthorship: A. Milne-Edwards, 1881; taxonomicStatus: accepted; **Location:** locationID: MRGID8403; waterBody: Pacific Ocean; country: Ecuador; stateProvince: Galapagos; locality: West; verbatimLocality: West of Fernandina; minimumDepthInMeters: 3407; maximumDepthInMeters: 3407; decimalLatitude: -0.3823; decimalLongitude: -91.8946; geodeticDatum: WGS84; coordinateUncertaintyInMeters: 100; **Identification:** identifiedBy: Mary Wicksten; dateIdentified: 2017; identificationRemarks: ID from imagery only; identificationQualifier: *Nematocarcinus* sp. indet.; **Event:** eventID: NA064; samplingProtocol: Remotely Operated Vehicles; eventDate: 07-03-15; eventTime: 11:56:38 AM; habitat: Lava Flow; **Record Level:** language: en; bibliographicCitation: WoRMS (2019). Nematocarcinus A. Milne-Edwards, 1881. Accessed at: http://www.marinespecies.org/aphia.php?p=taxdetails&id=107015 on 2019-08-23; institutionCode: CDF; collectionCode: Arthropoda; datasetName: Video transect framegrabs; basisOfRecord: HumanObservation

##### Notes

**Figure 29.**
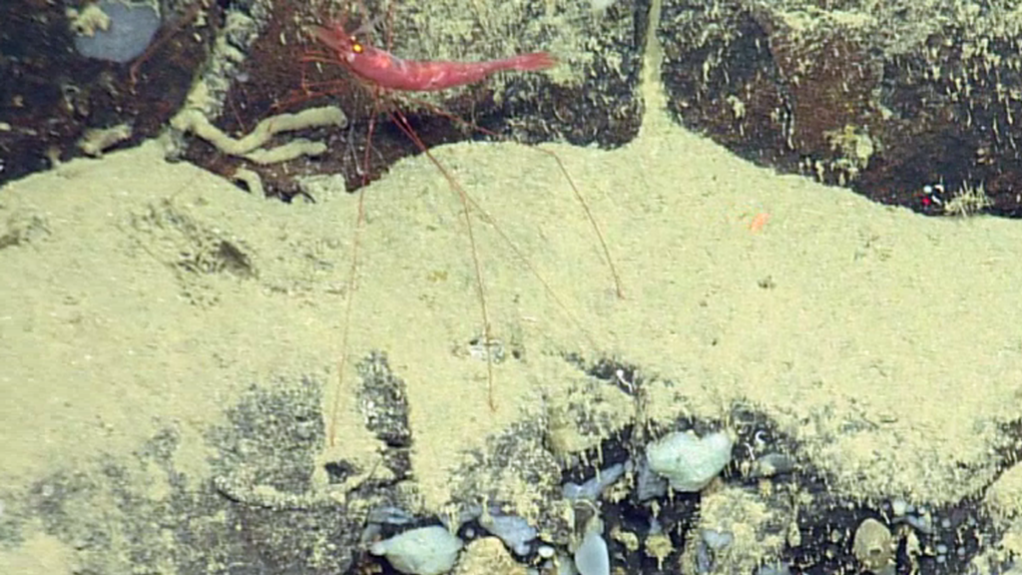
*Nematocarcinus* sp. indet.

The elongate, thread-like legs of this shrimp are easy to see in this photograph. However, as previously stated, the images were taken too far away to see features of the teeth on the rostrum or the relative length of the rostrum to the carapace and so neither Fig. 27 nor Fig. 28 can be identified beyond *Nematocarcinus*. Furthermore, it cannot be determined whether they are the same species or not. Fig. 29.

#### Pandaloidea

Haworth, 1825

5DA6A9CD-F3F2-5E37-815F-1E5FB00D5349

#### Plesionika trispinus

Squires & Barragan, 1976

1A8DFF12-F851-51DB-8303-CCE520417841

##### Materials

- **Type status:** Other material. **Occurrence:** recordedBy: CDF Volunteer; behavior: on seafloor; occurrenceStatus: present; preparations: Image only; associatedMedia: https://farm2.staticflickr.com/1974/31778746558_4a30c6259a_o.png; occurrenceID: H1443_024218_Plesionika_trispinus; **Taxon:** scientificNameID: urn:lsid:marinespecies.org:taxname:515523; scientificName: Plesionika trispinus; kingdom: Animalia; phylum: Arthropoda; class: Malacostraca; order: Decapoda; family: Pandalidae; genus: Plesionika; specificEpithet: trispinus; scientificNameAuthorship: Squires & Barragan, 1976; taxonomicStatus: accepted; **Location:** locationID: MRGID8403; waterBody: Pacific Ocean; country: Ecuador; stateProvince: Galapagos; locality: Southeast; verbatimLocality: Galapagos Platform; minimumDepthInMeters: 468; maximumDepthInMeters: 468; decimalLatitude: -0.3783; decimalLongitude: -90.8177; geodeticDatum: WGS84; coordinateUncertaintyInMeters: 15; **Identification:** identifiedBy: Mary Wicksten; dateIdentified: 2017; identificationRemarks: ID from imagery only; identificationQualifier: Plesionika trispinus; **Event:** eventID: NA064; samplingProtocol: Remotely Operated Vehicles; eventDate: 07-06-15; eventTime: 2:42:18 AM; habitat: Volcanic Cone; **Record Level:** language: en; bibliographicCitation: WoRMS (2019). Plesionika trispinus Squires & Barragan, 1976. Accessed at: http://www.marinespecies.org/aphia.php?p=taxdetails&id=515523 on 2019-08-23; institutionCode: CDF; collectionCode: Arthropoda; datasetName: Video transect framegrabs; basisOfRecord: HumanObservation

##### Notes

**Figure 30.**
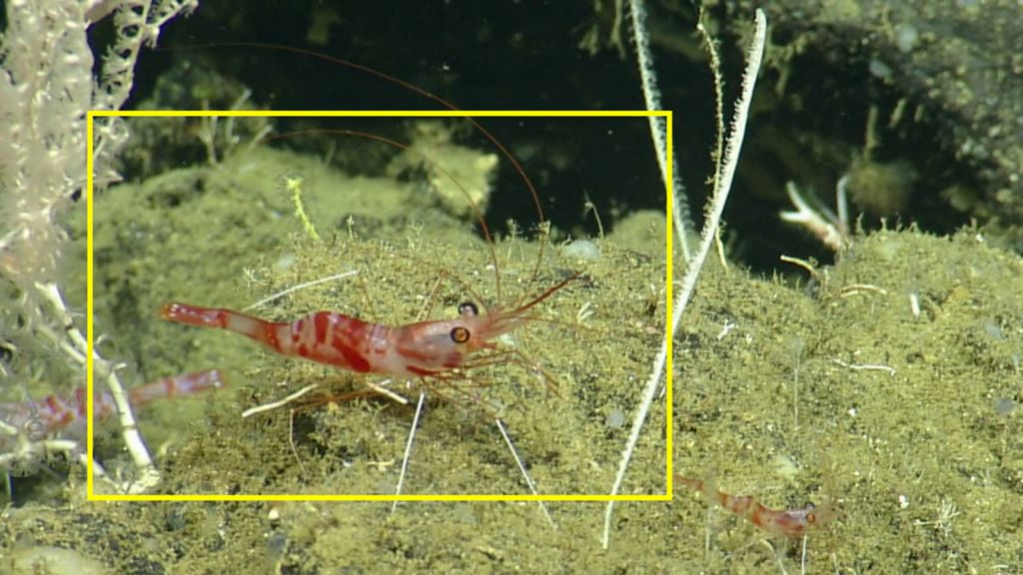
*Plesionika trispinus*

Key identifying features unclude rostrum and colour pattern. Fig. 30

#### Pandalidae

Haworth, 1825

2708C033-2630-5073-8CD3-6A10395668B3

##### Materials

- **Type status:** Other material. **Occurrence:** recordedBy: CDF Volunteer; behavior: in water column; occurrenceStatus: present; preparations: Image only; associatedMedia: https://farm2.staticflickr.com/1921/45600678172_5d263d61ed_o.png; occurrenceID: H1443_174932_Pandalidae_gen_inc_Heterocarpus; **Taxon:** scientificNameID: urn:lsid:marinespecies.org:taxname:106789; scientificName: Pandalidae; kingdom: Animalia; phylum: Arthropoda; class: Malacostraca; order: Decapoda; family: Pandalidae; scientificNameAuthorship: Haworth, 1825; taxonomicStatus: accepted; **Location:** locationID: MRGID8403; waterBody: Pacific Ocean; country: Ecuador; stateProvince: Galapagos; locality: Southeast; verbatimLocality: Galapagos Platform; minimumDepthInMeters: 638; maximumDepthInMeters: 638; decimalLatitude: -0.3796; decimalLongitude: -90.8107; geodeticDatum: WGS84; coordinateUncertaintyInMeters: 20; **Identification:** identifiedBy: Mary Wicksten; dateIdentified: 2017; identificationRemarks: ID from imagery only; identificationQualifier: *Heterocarpus* gen. inc; **Event:** eventID: NA064; samplingProtocol: Remotely Operated Vehicles; eventDate: 07-05-15; eventTime: 5:49:32 PM; habitat: Volcanic Cone; **Record Level:** language: en; bibliographicCitation: WoRMS (2019). Pandalidae Haworth, 1825. Accessed at: http://www.marinespecies.org/aphia.php?p=taxdetails&id=106789 on 2019-08-23; institutionCode: CDF; collectionCode: Arthropoda; datasetName: Video transect framegrabs; basisOfRecord: HumanObservation

##### Notes

**Figure 31.**
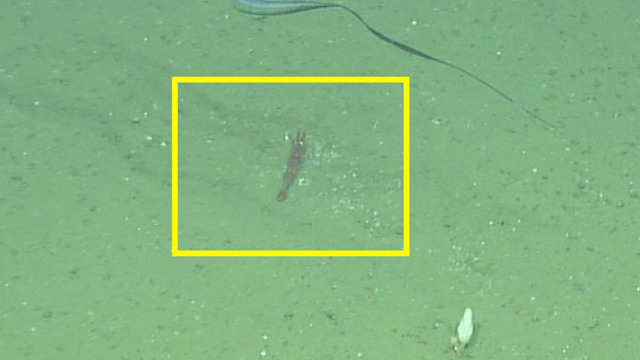
Pandalidae *Heterocarpus* gen. inc.

Robust body, living on mud, could indicate *Heterocarpus* gen. inc. Fig. 31

#### Pasiphaeoidea

Dana, 1852

64279A52-E138-5576-869D-425C2C82EA61

#### Pasiphaeidae

Dana, 1852

7799DF22-8381-5E59-A64B-266B19D7B20D

##### Materials

- **Type status:** Other material. **Occurrence:** recordedBy: CDF Volunteer; behavior: in water column; occurrenceStatus: present; preparations: Image only; associatedMedia: https://farm2.staticflickr.com/1922/31778742258_204d4c43a9_o.png; occurrenceID: H1443_190411_Pasiphaeidae_stet.; **Taxon:** scientificNameID: urn:lsid:marinespecies.org:taxname:106790; scientificName: Pasiphaeidae; kingdom: Animalia; phylum: Arthropoda; class: Malacostraca; order: Decapoda; family: Pasiphaeidae; scientificNameAuthorship: Dana, 1852; taxonomicStatus: accepted; **Location:** locationID: MRGID8403; waterBody: Pacific Ocean; country: Ecuador; stateProvince: Galapagos; locality: Southeast; verbatimLocality: Galapagos Platform; minimumDepthInMeters: 458; maximumDepthInMeters: 458; decimalLatitude: -0.375; decimalLongitude: -90.8144; geodeticDatum: WGS84; coordinateUncertaintyInMeters: 15; **Identification:** identifiedBy: Mary Wicksten; dateIdentified: 2017; identificationRemarks: ID from imagery only; identificationQualifier: Pasiphaeidae gen. indet.; **Event:** eventID: NA064; samplingProtocol: Remotely Operated Vehicles; eventDate: 07-05-15; eventTime: 7:04:11 PM; habitat: Volcanic Cone; **Record Level:** language: en; bibliographicCitation: WoRMS (2019). Pasiphaeidae Dana, 1852. Accessed at: http://www.marinespecies.org/aphia.php?p=taxdetails&id=106790 on 2019-08-23; institutionCode: CDF; collectionCode: Arthropoda; datasetName: Video transect framegrabs; basisOfRecord: HumanObservation

##### Notes

**Figure 32.**
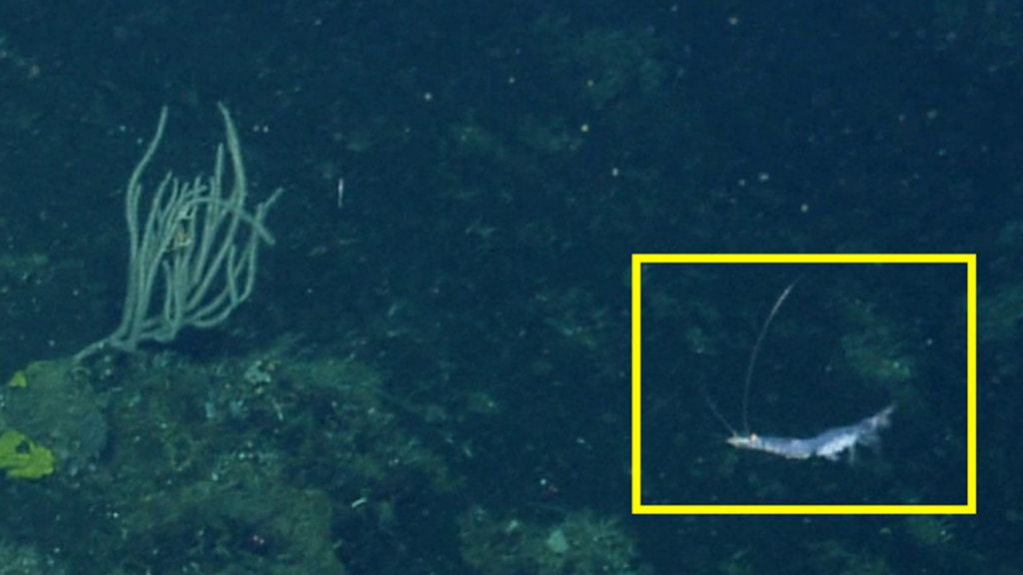
Pasiphaeidae gen. indet.

Translucent, carries eggs so must be Caridea, elongate body, however, cannot see legs to be sure. Fig. 32

#### Polychelida

Scholtz & Richter, 1995

F4679A18-45AC-583F-8CF1-0BB280F89DE3

#### Pentacheles laevis

Bate, 1878

D0643541-CFBE-51FE-9B88-5C31C889BF53

##### Materials

- **Type status:** Other material. **Occurrence:** recordedBy: CDF Volunteer; behavior: in water column; occurrenceStatus: present; preparations: Image only; associatedMedia: https://farm2.staticflickr.com/1928/30710128297_f510ddf041_o.png; occurrenceID: H1440_084149_Pentacheles_laevis; **Taxon:** scientificNameID: urn:lsid:marinespecies.org:taxname:382979; scientificName: Pentacheles laevis; kingdom: Animalia; phylum: Arthropoda; class: Malacostraca; order: Decapoda; family: Polychelidae; genus: Pentacheles; specificEpithet: laevis; scientificNameAuthorship: Bate, 1878; taxonomicStatus: accepted; **Location:** locationID: MRGID8403; waterBody: Pacific Ocean; country: Ecuador; stateProvince: Galapagos; locality: Far North; verbatimLocality: Northwest of Darwin; minimumDepthInMeters: 1336; maximumDepthInMeters: 1336; decimalLatitude: 1.8838; decimalLongitude: -92.1331; geodeticDatum: WGS84; coordinateUncertaintyInMeters: 40; **Identification:** identifiedBy: Mary Wicksten; dateIdentified: 2017; identificationRemarks: ID from imagery only; identificationQualifier: Pentacheles laevis; **Event:** eventID: NA064; samplingProtocol: Remotely Operated Vehicles; eventDate: 07-02-15; eventTime: 8:41:49 AM; habitat: Seamount; **Record Level:** language: en; bibliographicCitation: WoRMS (2019). Pentacheles laevis Bate, 1878. Accessed at: http://www.marinespecies.org/aphia.php?p=taxdetails&id=382979 on 2019-08-23; institutionCode: CDF; collectionCode: Arthropoda; datasetName: Video transect framegrabs; basisOfRecord: HumanObservation

##### Notes

**Figure 33.**
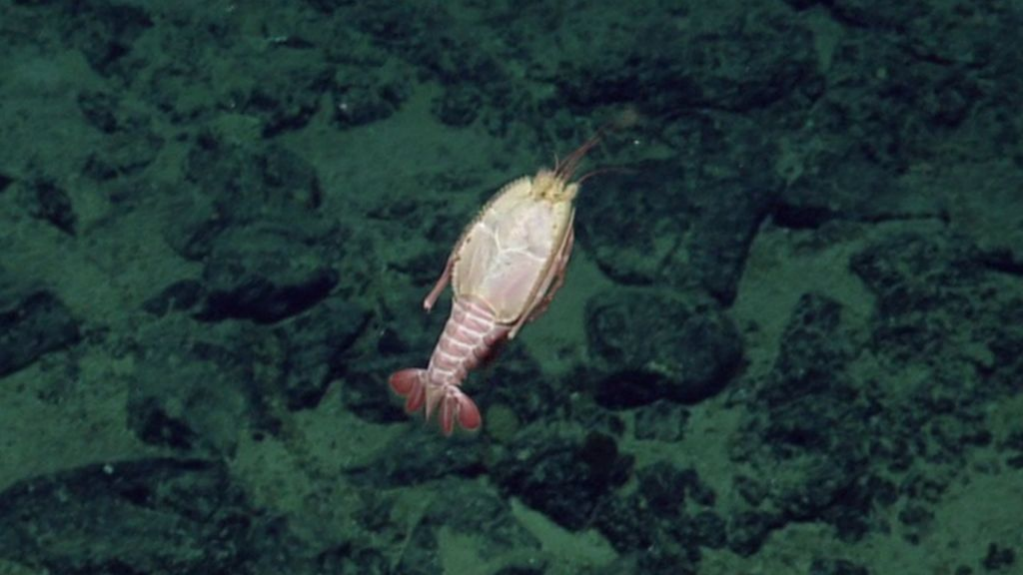
*Pentacheles laevis*

See discussion for more detailed comments on this observation. Fig. 33

## Discussion

Here we provide the first and most complete image inventory of arthropods found in the deep waters of the GMR to date. Of particular interest was the presence of three species that are new records to the GMR; *Sternostylus defensus, Tylaspis anomala and Paromola rathbunae* sp. inc. (Figs 7, 22, 25), in-situ imagery of two new species recently described, *Heteroptychus nautilus* and *Eumunida subsolanus* (Baba and Wicksten 2019) (Figs 3, 6) and at least one species of squat lobster that is possibly new to science, *Sternostylus* sp. indet. (Fig. 8). Based on the occurrences presented here, these morphospecies could be targeted on future expeditions.

The species of *Eumunida subsolanus* (Fig. 6) and the unidentified mathildellid crab (Fig. 24), to the best of our knowledge, represent the first records of their respective families for the eastern Pacific and the latter is also the first record for the family Eumunididae in the Tropical Eastern Pacific (Baba and Wicksten 2019). The mathildellid crab appears to represent either *Mathildella* or *Neopilumnoplax*, species of which have been previously recorded from seamounts and deep-sea ridges in the Indo-West Pacific and tropical Atlantic (Ahyong 2008, Ahyong and Ng 2016).

Overall, we observed many different types of arthropod behaviour and associations. For example, we found species from the genera *Heteroptychus, Uroptychus, Eumunida* and *Sternostylus* displaying a preference for gorgonians and black corals as hosts (Figs 3, 4, 5, 6, 7, 8) (Buhl-Mortensen and Mortensen 2004, Clark et al. 2010, Guilloux et al. 2010, Baillon et al. 2014). This also occurred in shrimps, such as *Bathypalaemonella* sp. indet. usually found in lush sponge and coral gardens (Fig. 27). This mutualistic relationship consists of the arthropods gaining food, habitat and protection from potential predators, while enhancing coral survival by cleaning them from sediment particles (Buhl-Mortensen and Mortensen 2004, Guilloux et al. 2010).

We also recorded two rarely-observed deep-water hermit crabs *Probeebei mirabilis* and *Tylaspis anomala* (*Figs 21, 22*), the latter displaying the behaviour of carrying a sea anemone as observed for specimens from New Caledonia (Lemaitre 1998). The hermit crab-anemone mutualistic relationship is very common in both shallow and deep-sea environments (Hazlett 1981, Jonsson et al. 2001, Mercier and Hamel 2008). *Tylaspis anomala* was recorded for the first time at the GMR (Fig. 22).

The brachyuran crab *Paromola rathbunae* sp. inc. was found carrying a sponge using its fifth pereiopods (Fig. 25a, b). This is commonly observed in several crustacean families, including homolids; however, most of these reports are from the Atlantic Ocean (Braga-Henriques et al. 2011, Capezzuto et al. 2012). *Paromola* is well documented from sponge and coral gardens (Capezzuto et al. 2012). In the *Nautilus* expedition, we found a large aggregation of homolid crabs all carrying different shaped-sponges (Fig. 25c, d). To our knowledge, a large group of homolid crabs, such as the one presented here, has not been previously reported for the Tropical Eastern Pacific (Muñoz et al. 2011, Smith et al. 2011, Hendrickx and Wicksten 2016). *Paromola rathbunae* sp. inc. is also a new record for the GMR.

The polychelid lobster, *Pentacheles laevis* (Fig. 33), although already recorded from the Galapagos Islands (Galil 2000), was observed for the first time in-situ. Such observations of Polychelidae are rare and, to date, individuals have been observed only on the surface of, or more often, buried in the substrate (Ahyong 2009). Our observations of *P. laevis* actively swimming were the first such records for the family. The observed individual was not engaged in the typical ‘caridoid’ escape behaviour (i.e. rapid backward propulsion by pleonic flexion) of most decapods. Instead, the polychelid was swimming forwards using the pleopods and with a straightened pleon in typical ‘natantian’ fashion – a plesiomorphic trait not exhibited by other lobsters and crayfish or most other reptant decapods. Although the polychelids had long been placed amongst the achelate lobsters (Palinura), the ‘natantian’ swimming mode, observed here, is consistent with the basal position of polychelids amongst reptantian decapods as determined by many phylogenetic analyses (e.g. Scholtz 1995, Dixon et al. 2003, Ahyong and O’Meally 2004, Bracken-Grissom et al. 2014).

Since the 1950s, fisheries have been shifting towards deeper waters in most parts of the world, threatening deep-sea biodiversity and resulting in over-exploitation of seamount faunas (Morato Gomes and Pauly 2004, Morato et al. 2006, Clark et al. 2010). As a result, the last havens for commercial species are being exploited and, with only little over 4% of the world’s deep-sea having been studied (Morato Gomes and Pauly 2004), we might be losing species that are still waiting to be discovered (Mora et al. 2011). This study highlights the importance of assessing deep-sea communities within protected areas and we strongly emphasise the need to publish taxonomic inventories, since these studies serve as a reference for understanding species ecology and for developing future conservation measures.

## Supplementary Material

XML Treatment for Dendrobranchiata

XML Treatment for Penaeoidea

XML Treatment for Pleocyemata

XML Treatment for Anomura

XML Treatment for Chirostyloidea

XML Treatment for Heteroptychus nautilus

XML Treatment for Uroptychus bellus

XML Treatment for Uroptychus

XML Treatment for Eumunida subsolanus

XML Treatment for Sternostylus defensus

XML Treatment for Sternostylus

XML Treatment for Galatheoidea

XML Treatment for Janetogalathea

XML Treatment for Munida

XML Treatment for Munida

XML Treatment for Munidopsis albatrossae

XML Treatment for Munidopsis hystrix

XML Treatment for Munidopsis mina

XML Treatment for Munidopsis

XML Treatment for Munidopsis

XML Treatment for Munidopsis

XML Treatment for Munidopsis

XML Treatment for Lithodoidea

XML Treatment for Lithodes panamensis

XML Treatment for Paralomis

XML Treatment for Paguroidea

XML Treatment for Probeebei mirabilis

XML Treatment for Tylaspis anomala

XML Treatment for Brachyura

XML Treatment for Cancroidea

XML Treatment for Metacarcinus

XML Treatment for Goneplacoidea

XML Treatment for Mathildellidae

XML Treatment for Homoloidea

XML Treatment for Paromola

XML Treatment for Majoidea

XML Treatment for Lophorochinia parabranchia

XML Treatment for Caridea

XML Treatment for Campylonotoidea

XML Treatment for Bathypalaemonella

XML Treatment for Nematocarcinoidea

XML Treatment for Nematocarcinus

XML Treatment for Nematocarcinus

XML Treatment for Pandaloidea

XML Treatment for Plesionika trispinus

XML Treatment for Pandalidae

XML Treatment for Pasiphaeoidea

XML Treatment for Pasiphaeidae

XML Treatment for Polychelida

XML Treatment for Pentacheles laevis

## Figures and Tables

**Figure 6a.**
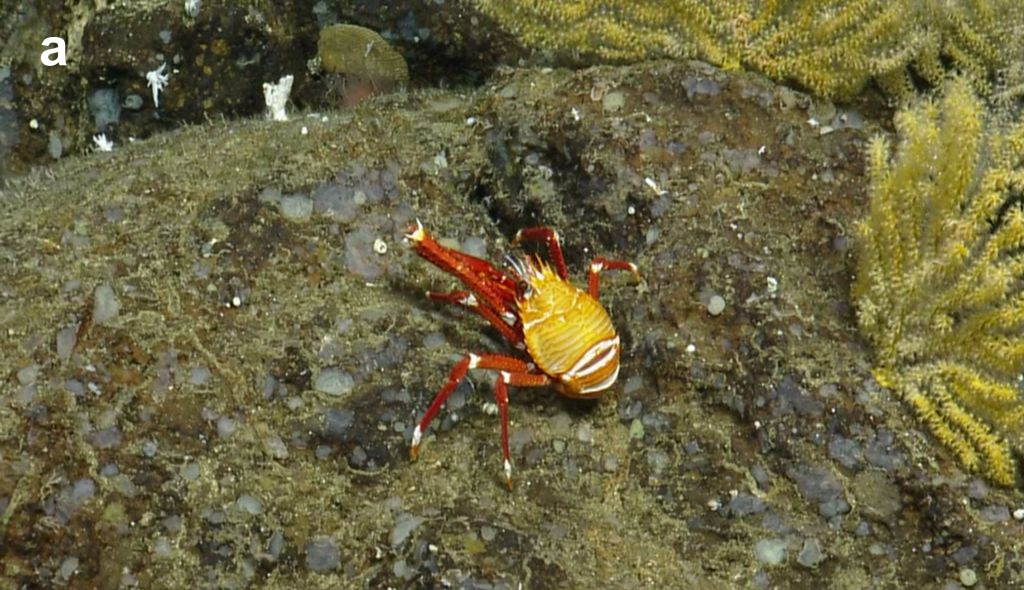
Single *Eumunida subsolanus* on a rock.

**Figure 6b.**
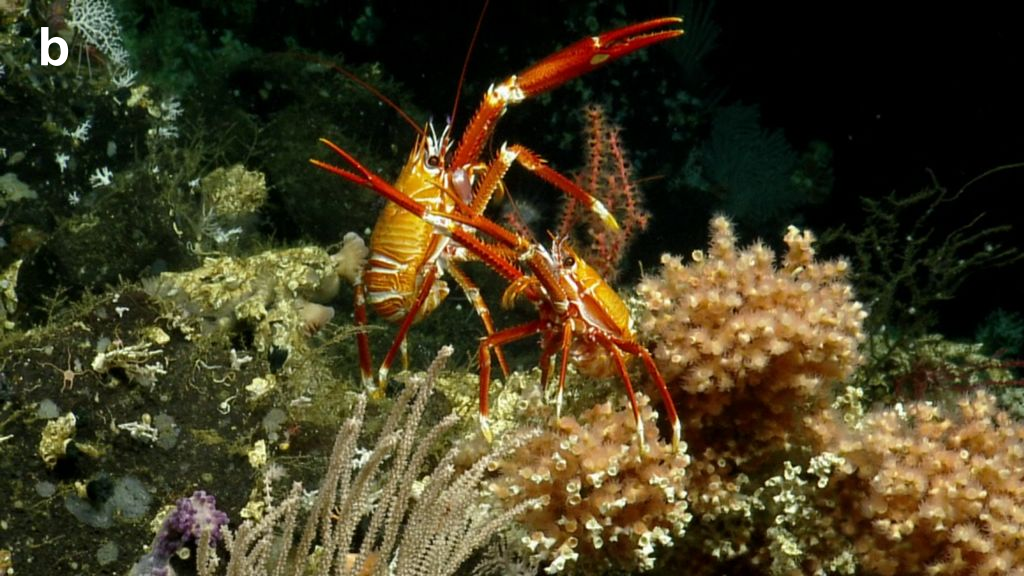
Two *Eumunida subsolanus* amongst a coral garden.

**Figure 6c.**
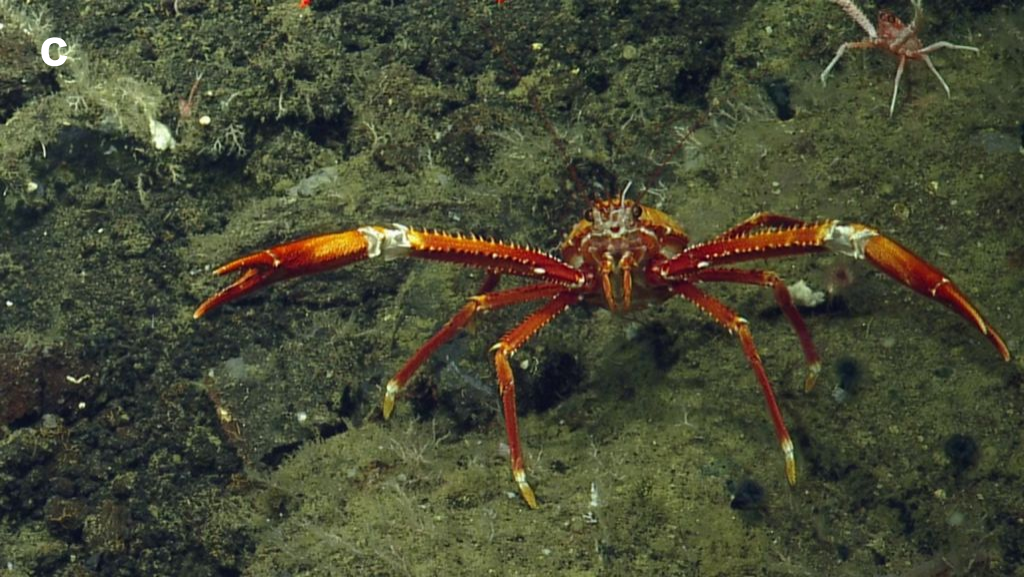
Single *Eumunida subsolanus*.

**Figure 6d.**
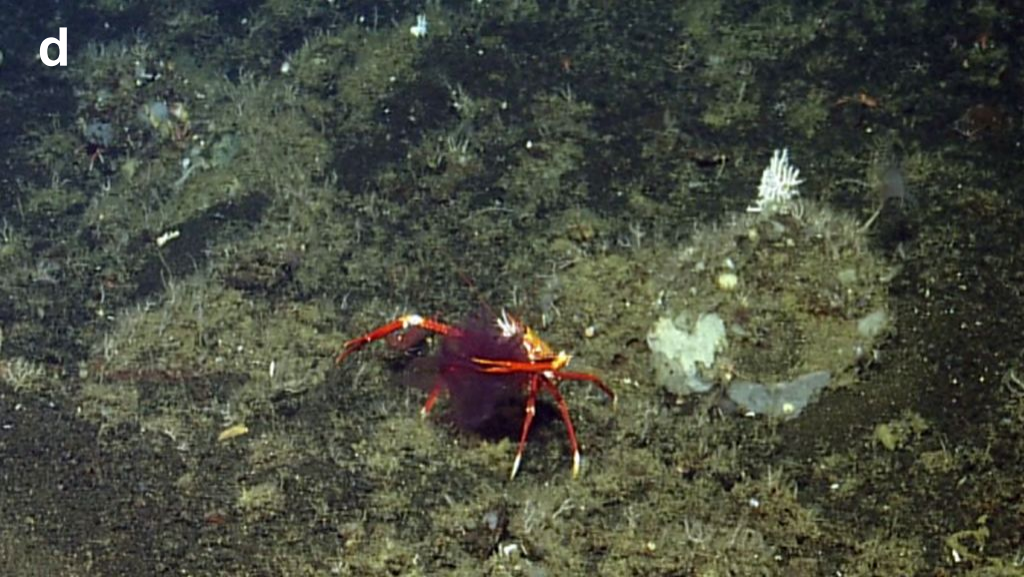
*Eumunida subsolanus* consuming prey.

**Figure 13a.**
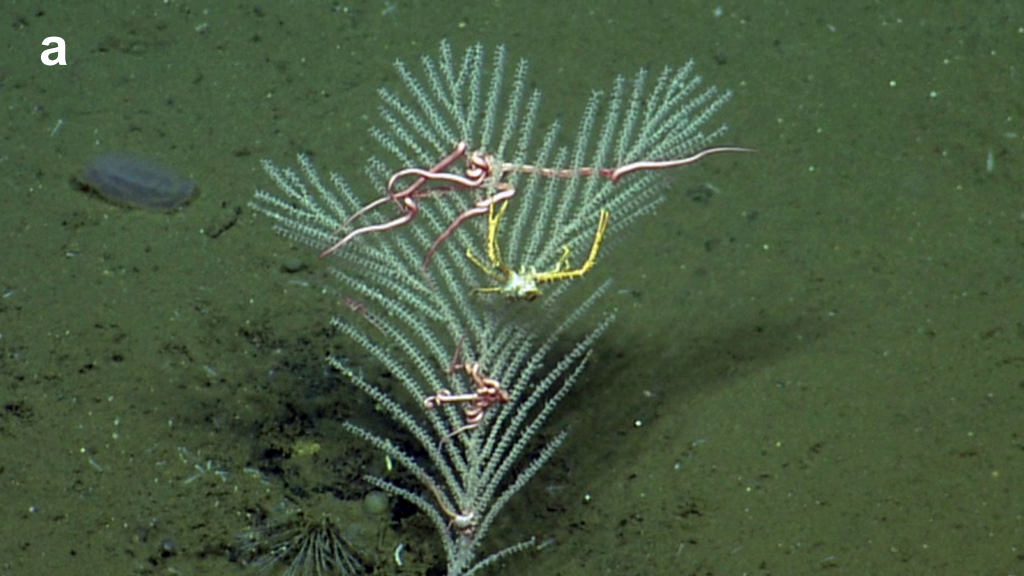
*Munidopsis hystrix* on octocoral.

**Figure 13b.**
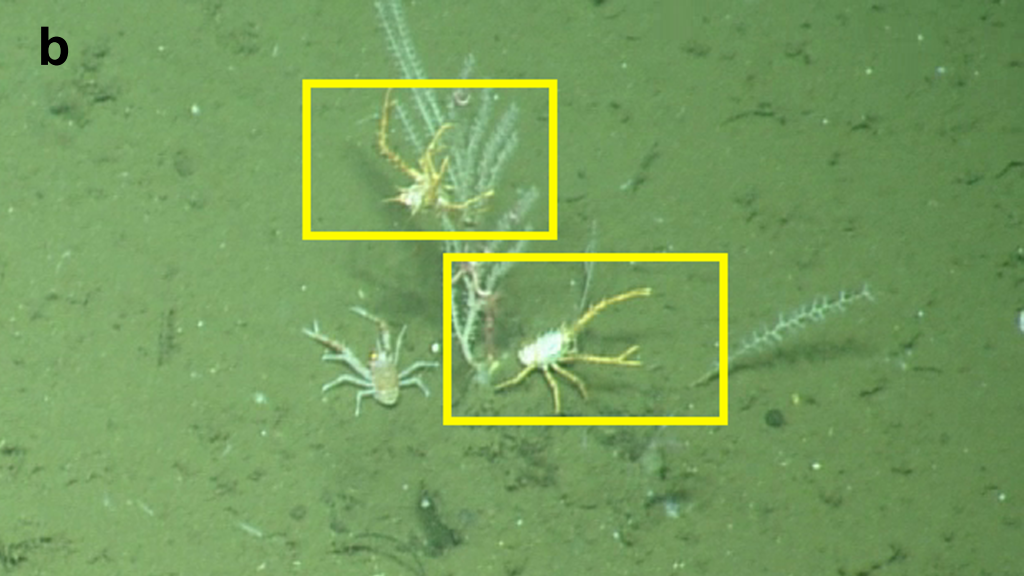
Two *Munidopsis hystrix.*

**Figure 24a.**
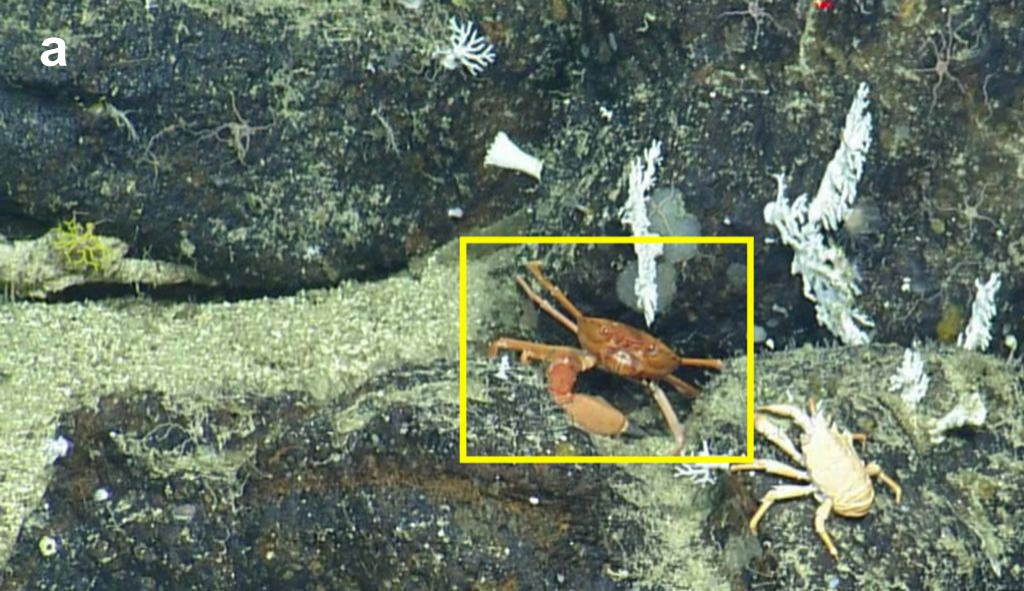
Mathildellidae gen. indet. on rocks.

**Figure 24b.**
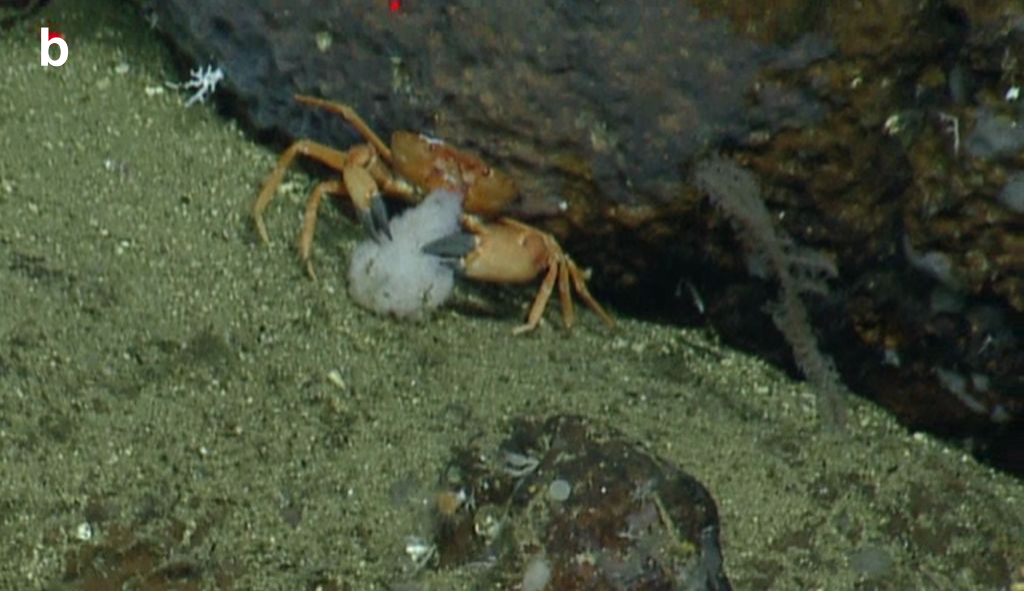
Mathildellidae gen. indet. feeding on a sponge.

**Figure 25a.**
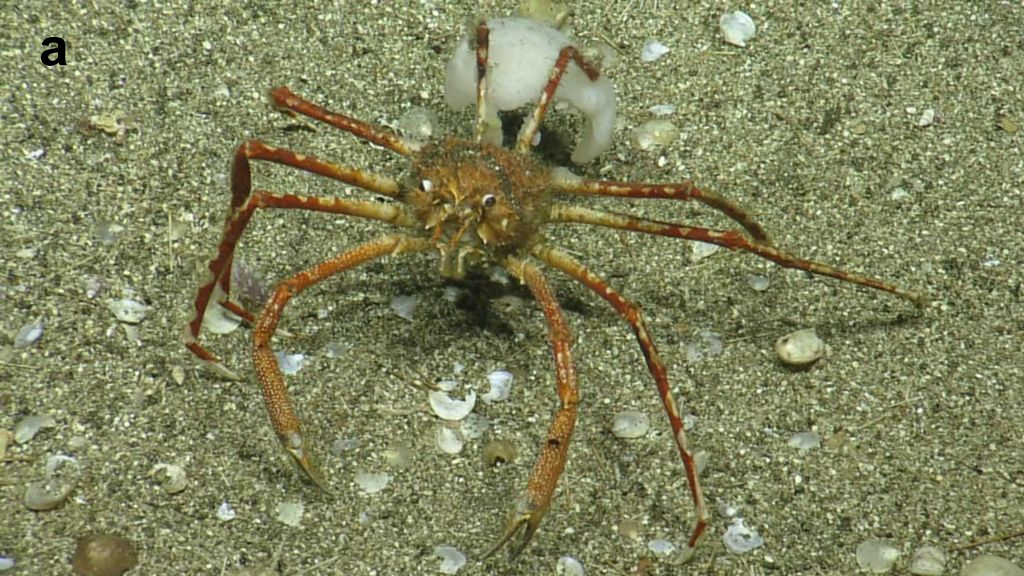
Single *Paromola rathbunae* sp. inc. with a sponge.

**Figure 25b.**
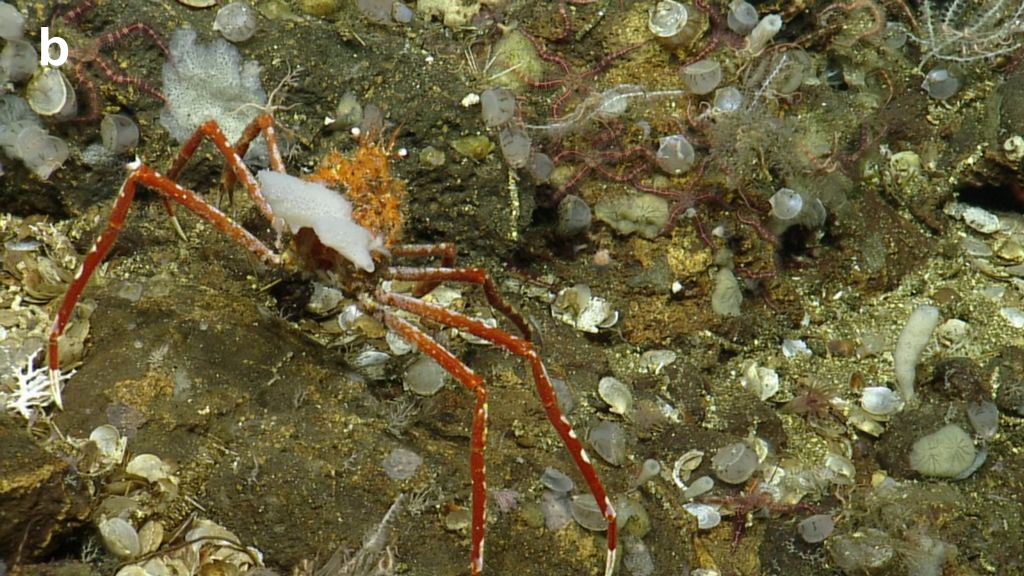
Single *Paromola rathbunae* sp. inc.

**Figure 25c.**
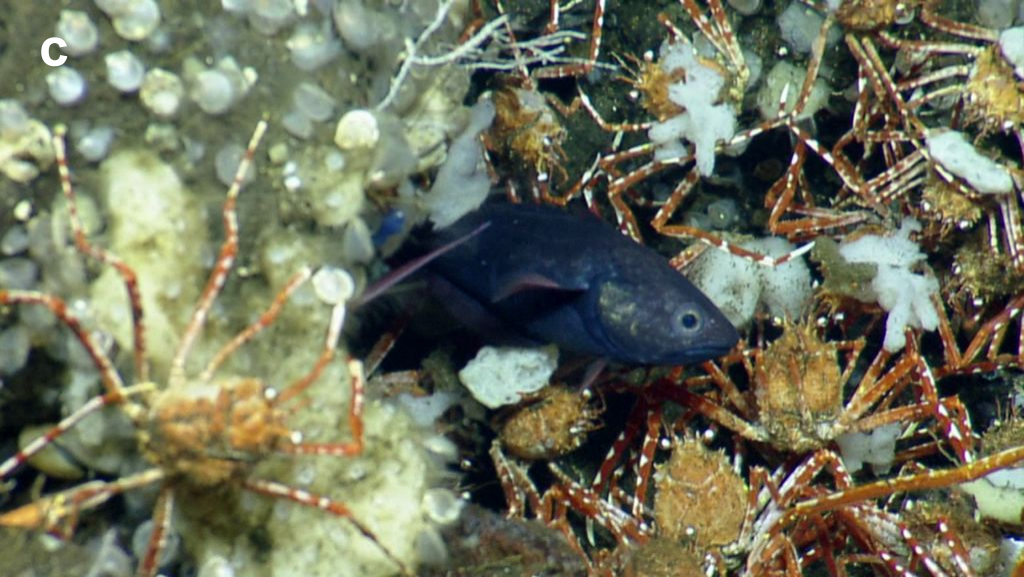
Aggregation of *Paromola rathbunae* sp. inc.

**Figure 25d.**
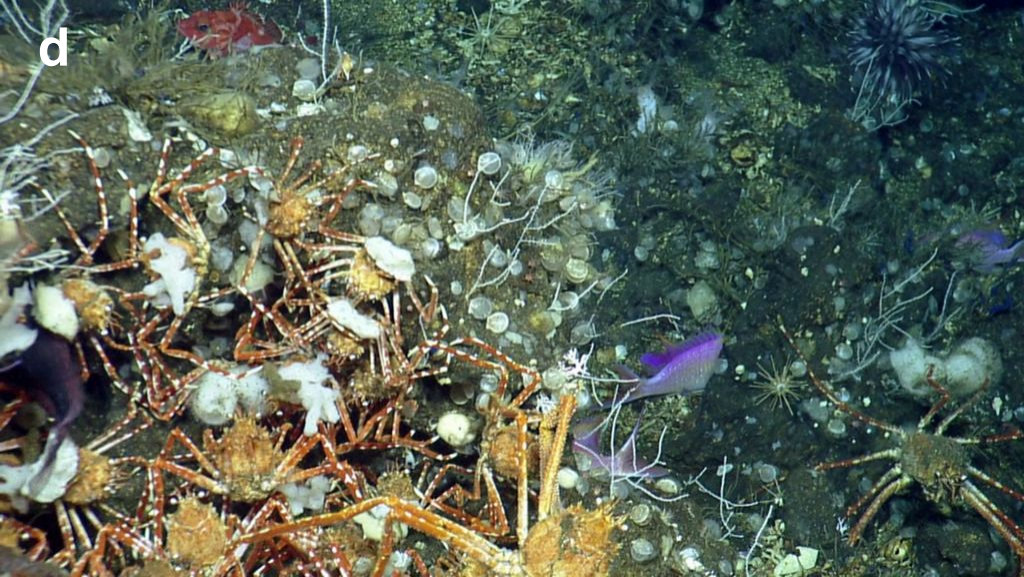
Aggregation of *Paromola rathbunae* sp. inc.

**Table 1.** EV *Nautilus* Sampling sites details.

**Dive**	**Geographical location**	**Feature**	**Depth range (m)**	**ROV Bottom time**	**Latitude, Longitude**
H1435	East of Wolf	Seamount	1120-290	16 h 48	1.2105, -91.0836
H1436	East of Darwin	Seamount	2090-930	11 h 14	1.6584, -91.6731
H1440	North of Darwin	Seamount	1960-1190	11 h 21	1.8535, -92.1064
H1441	West of Fernandina	Lava flows	3370-3300	13 h 34	-0.3763, -91.9043
H1442	West of Fernandina	Lava flows	3010-2940	18 h 25	-0.3763, -91.7619
H1443	West of Santiago	Seamount	640-250	18 h 16	-0.3824, -90.8091
